# New perspectives on the plant PARP family: *Arabidopsis* PARP3 is inactive, and PARP1 exhibits predominant poly (ADP-ribose) polymerase activity in response to DNA damage

**DOI:** 10.1186/s12870-019-1958-9

**Published:** 2019-08-19

**Authors:** Zongying Gu, Weiyang Pan, Wei Chen, Qichao Lian, Qiao Wu, Zeyu Lv, Xuan Cheng, Xiaochun Ge

**Affiliations:** State Key Laboratory of Genetic Engineering, Department of Biochemistry and Molecular Biology, School of Life Sciences, Fudan University, Shanghai, 200438 China

**Keywords:** *Arabidopsis thaliana*, DNA damage response, ADP-ribosylation, Posttranslational modification, Poly (ADP-ribose) polymerase

## Abstract

**Background:**

Poly (ADP-ribosyl) ation (PARylation) is an important posttranslational modification that regulates DNA repair, gene transcription, stress responses and developmental processes in multicellular organisms. Poly (ADP-ribose) polymerase (PARP) catalyzes PARylation by consecutively adding ADP-ribose moieties from NAD^+^ to the amino acid receptor residues on target proteins. *Arabidopsis* has three canonical PARP members, and two of these members, AtPARP1 and AtPARP2, have been demonstrated to be bona fide poly (ADP-ribose) polymerases and to regulate DNA repair and stress response processes. However, it remains unknown whether AtPARP3, a member that is highly expressed in seeds, has similar biochemical activity to that of AtPARP1 and AtPARP2. Additionally, although both the phylogenetic relationships and structural similarities indicate that AtPARP1 and AtPARP2 correspond to animal PARP1 and PARP2, respectively, two previous studies have indicated that AtPARP2, and not AtPARP1, accounts for most of the PARP activity in *Arabidopsis*, which is contrary to the knowledge that PARP1 is the predominant PARP in animals.

**Results:**

In this study, we obtained both in vitro and in vivo evidence demonstrating that AtPARP3 does not act as a typical PARP in *Arabidopsis*. Domain swapping and point mutation assays indicated that AtPARP3 has lost NAD^+^-binding capability and is inactive. In addition, our results showed that AtPARP1 was responsible for most of the PARP enzymatic activity in response to the DNA damage-inducing agents zeocin and methyl methanesulfonate (MMS) and was more rapidly activated than AtPARP2, which supports that AtPARP1 remains the predominant PARP member in *Arabidopsis*. AtPARP1 might first become activated by binding to damaged sites, and AtPARP2 is then poly (ADP-ribosyl) ated by AtPARP1 in vivo.

**Conclusions:**

Collectively, our biochemical and genetic analysis results strongly support the notion that AtPARP3 has lost poly (ADP-ribose) polymerase activity in plants and performs different functions from those of AtPARP1 and AtPARP2. AtPARP1, instead of AtPARP2, plays the predominant role in PAR synthesis in both seeds and seedlings. These data bring new insights into our understanding of the physiological functions of plant PARP family members.

**Electronic supplementary material:**

The online version of this article (10.1186/s12870-019-1958-9) contains supplementary material, which is available to authorized users.

## Background

Poly (ADP-ribose) polymerase (PARP), the enzyme that catalyzes the protein poly (ADP-ribosyl) ation (PARylation) modification, exists widely in different eukaryotes except yeast [1]. PARP transfers ADP-ribose units from the substrate NAD^+^ to acceptor amino acids on target proteins to produce long linear or branched chains of poly (ADP-ribose) (PAR), and these units can then be removed by a hydrolysis enzyme, poly (ADP-ribose) glycohydrolase (PARG) [1, 2]. Modified target proteins include the enzyme itself, histones, DNA repair proteins, transcription factors, chromatin modulators [2, 3] and proteins involved in defense response [4]. Due to its highly negative charges, PAR largely affects the protein-protein interaction features of target proteins. PAR might act as a protein-binding scaffold to recruit other proteins for the formation of large complexes [2]. Proteins can bind to PARylated proteins through their PAR-binding motifs [2, 5]. PARP family members have been extensively studied in humans because they are implicated in multiple pathogenesis processes [6, 7]. PARP inhibitors for use in research or medicine have then been developed, and some of these mimic the structure of NAD^+^ and act as competitive inhibitors, such as 3-aminobenzamide (3-AB) [5, 8–10]. Seventeen members in humans are involved in various physiological processes, such as DNA repair, cell death, transcriptional regulation, energy metabolism, and chromatin remodeling [2, 11, 12].

The functions of the PARP family in plants are much less well understood. PARP activities have been identified in the nuclei of different species, such as wheat [13], pea [13], soybean [14], tobacco [15, 16], maize [13, 17] and *Arabidopsis* [17]. To date, three types of PARPs with different molecular masses and structural architectures have been found in plants. In *Arabidopsis,* these PARPs are named AtPARP1, AtPARP2 and AtPARP3 [18, 19]. AtPARP1 and AtPARP2 have auto-PARylation activity in vitro and in vivo [17, 20–22], and AtPARP2 is the predominant PARP in DNA damage and immune responses [22], but whether AtPARP3 has PARP activity remains elusive. AtPARP1 and AtPARP2 have been shown to regulate genotoxic [23, 24], biotic and abiotic stress responses [4, 10, 14, 15, 20, 22, 25, 26], as well as leaf and root development [9, 21, 27, 28]. However, a recent study indicated that double and triple mutants of PARP family members have no phenotypes under abiotic stresses or under biotic stress stimulated by the microbe-associated molecular pattern (MAMP) flg22 [29], and as a result, the in vivo functions of PARPs in both biotic and abiotic stresses are controversial. *AtPARP3* is highly expressed in seeds, where both *AtPARP1* and *AtPARP2* are almost undetectable [30, 31]. It is reported that AtPARP3 is involved in the maintenance of seed viability during seed storage [27, 30], therefore, AtPARP3, instead of AtPARP1 and AtPARP2, was hypothesized to be the major enzyme catalyzing PARylation and responsible for DNA repair during seed germination [30, 31].

PARP proteins are classified by the PARP signature consisting of a central six-stranded β sheet that binds and catalyzes the decomposition of NAD^+^ [11, 19, 32]. A catalytic core motif histidine-tyrosine-glutamic acid (H-Y-E) triad has been found in all active PARP proteins and is involved in NAD^+^ binding. Based on the domain architecture of the proteins, AtPARP1 and AtPARP2 resemble human HsPARP1 and HsPARP2, respectively [18, 19], whereas AtPARP3 has no counterpart in humans.

Whether a protein exhibits known biochemical activity is important for explaining its physiological role. To understand the function of AtPARP3 in seeds, it is critical to determine its enzymatic activity. In addition, elucidating the functional relationships among members of this protein family will also help us understand the family’s evolution and differentiation. In this study, through a combination of biochemical and genetic approaches, we demonstrated that AtPARP3 exhibits no poly (ADP-ribosyl) ation activity in vitro and in vivo. We also found that AtPARP1 exhibits predominant PARP enzymatic activity relative to other family members in both seeds and seedlings when stimulated by DNA breaks. This result differs from the previous finding that AtPARP2 is the predominant PARP member in DNA damage response but is consistent with the role of its animal PARP1 counterpart, which accounts for most of the intracellular PARP activity under genotoxic stress. Our results shed new light on the nature of the plant PARP family.

## Results

### Domain architectures and phylogenetic relationships of PARP1/2/3

AtPARP1 is very similar to HsPARP1 in terms of its domain architecture (Additional file 1: Figure S1A). HsPARP1 is thought to be the founding member of the PARP family in humans [1, 3, 11]. Both HsPARP1 and AtPARP1 have five important domains with known functions arranged from the N to the C terminus: three N-terminal zinc fingers responsible for DNA damage detection, the BRCA-1 C-terminus (BRCT) domain for phospho-protein binding, the WGR domain with the conserved Trp-Gly-Arg (WGR) motif for nucleic acid binding, the PARP regulatory domain (PRD) or helical subdomain (HD) believed to regulate PAR-branching, and the C-terminal characteristic PARP domain with catalytic activity [3, 33] (Additional file 1: Figure S1A). Both HsPARP2 and AtPARP2 have no zinc fingers or BRCT domain but do have two SAF/Acinus/PIAS motif (SAP) domains in the N-terminal region that confer DNA-binding activity [11, 19, 32]. HsPARP3 has only three domains, i.e., the WGR, PRD/HD and PARP domains, and catalyzes mono (ADP-ribosyl) ation (mART), which attaches only one ADP-ribose unit to the target protein [34], whereas AtPARP3 has a long N-terminal region with unknown functions and bears BRCT, WGR, PRD/HD and the PARP catalytic domain.

The sequence analysis revealed that both AtPARP1 and AtPARP2 have a typical H-Y-E catalytic triad, whereas AtPARP3 has an alternative histidine-valine-glutamic acid (C-V-E) triad in its catalytic core (Additional file 1: Figure S1B). To understand the evolutionary relationships of the PARP family in different species, we constructed a phylogenetic tree using PARP1/2/3 homologs identified from twenty-eight organisms, including eighteen angiosperms, two gymnosperms, one lycopod, one moss, four metazoans and two fungi, using the PARP1 subfamily as an outgroup (Additional file 2: Figure S2). The phylogenetic tree topology revealed that the PARP1/2/3 subfamilies form two clades in eukaryotes, each with metazoan members, which suggests that the clade containing plant PARP2 and the clade with plant PARP1/3 originated from gene duplications before the common ancestor of eukaryotic organisms. The close homologs of plant PARP1 and PARP3 form separate phylogenetic groups, each involving lycopod and moss members, which indicates that these resulted from duplications before the divergence of extant land plants but after the separation of plants from metazoans and fungi.

### AtPARP3 does not show auto-ADP-ribosylation activity in vitro

**Fig. 1:**
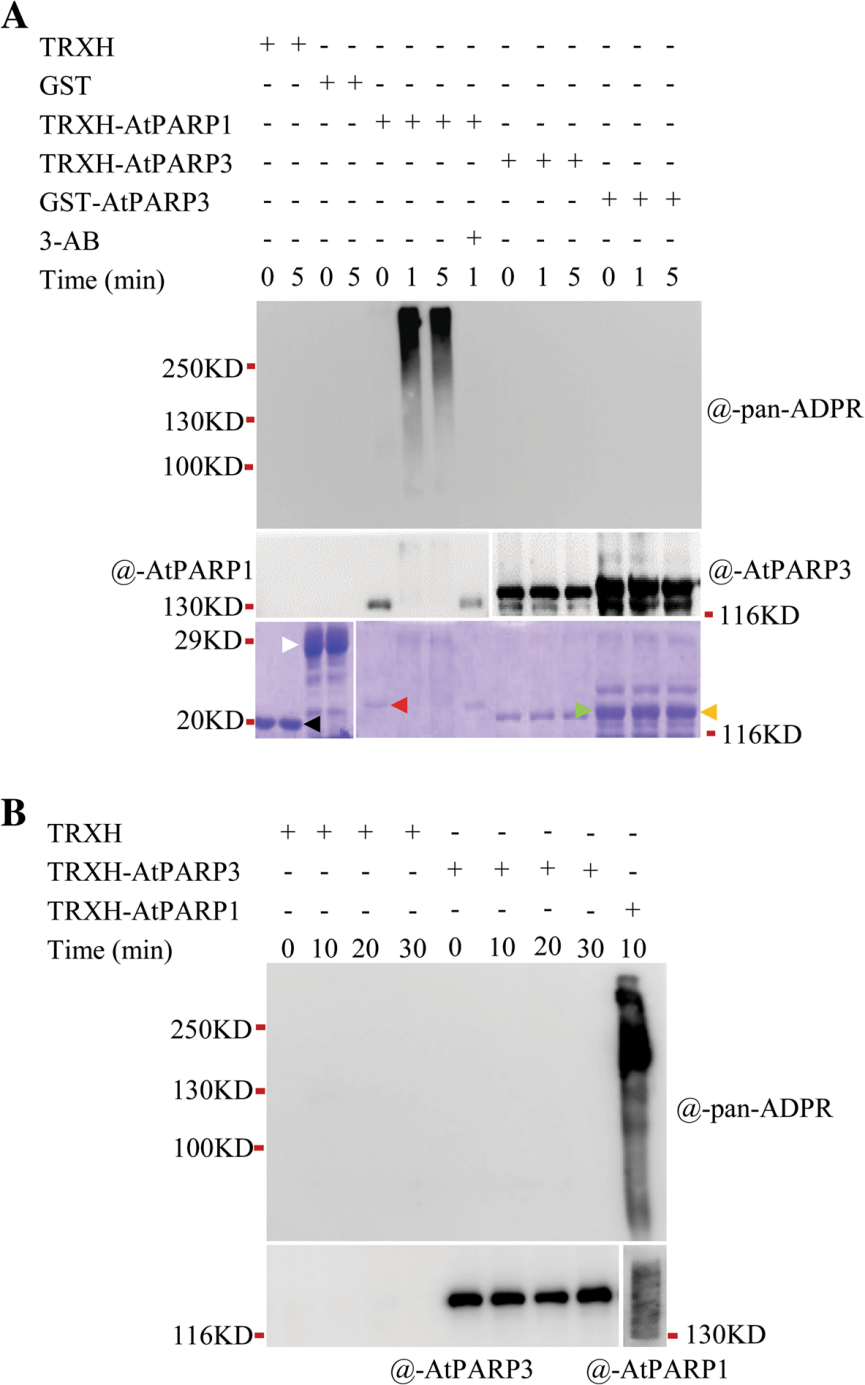
Determination of the enzymatic activity of AtPARP3. **a** Recombinant AtPARP3 activity determination. The purified proteins were incubated with 500 nM broken DNA and 1 mM NAD^+^ at 25 °C for different time periods with or without 20 mM 3-AB. After electrophoresis, immunoblotting was performed with anti-pan-ADPR reagent or other internal control antibodies. The tag proteins, TRXH and GST, expressed by the empty vectors pET32a(+) and pGEX-4 T-1, respectively, were used as the negative controls for the assay. The Coomassie blue-stained SDS-PAGE gel in the bottom panel shows the loading amounts of TRXH (black arrow), GST (white arrow), TRXH-AtPARP1 (red arrow), TRXH-AtPARP3 (green arrow), and GST-AtPARP3 (orange arrow). **b** Activity determination under extended time periods. The purified proteins were incubated with 500 nM broken DNA and 1 mM NAD^+^ at 25 °C for different time periods. The poly (ADP-ribosyl) ated proteins were separated on an SDS-PAGE gel and detected by anti-pan-ADPR reagent (the upper panel) and anti-AtPARP3 and anti-AtPARP1 antibodies, respectively

Because the activity of AtPARP3 is unknown, we mainly focused on AtPARP3. We first examined the biochemical activity of recombinant AtPARP3 protein expressed in *E. coli*. To avoid the possible influence of a fusion tag on protein function, we used two different expression vectors, pGEX-4 T-1 and pET-32a(+), which carry a glutathione S-transferase (GST) and a thioredoxin and histidine (TRXH) tag fused to the N terminus of the recombinant protein, respectively. These two tags reportedly facilitate the correct folding of the target proteins, particularly the cysteine-rich proteins. AtPARP1 was expressed by the pET-32a(+) vector as a positive control for the activity assay because it shows stronger in vitro activity than AtPARP2, as demonstrated in our previous studies [21]. The tag proteins were also expressed using the empty vectors for use as negative controls. We assessed their activities through a standard PARP activity assay based on the auto-modification feature of PARP proteins [35]. A sensitive ADP-ribose detection reagent that can detect both mono- and poly-ADP-ribose, called anti-pan-ADP-ribose binding reagent, was used to detect the ADP-ribose moiety on proteins (based on the certificate of analysis provided by Merck). It has been successfully used in a prior study on humans [36]. As shown in Fig. 1a, a strong PAR signal was generated by AtPARP1 within 1 min, and the band exhibited an upward smear typical of poly (ADP-ribosyl) ation, consistent with previous observations that PARP1 is a robust enzyme synthesizing PAR in seconds [35, 37]. The signal was abolished by the PARP inhibitor 3-AB, which indicated that it was a real PAR signal. However, no ADP-ribose signal was detected in the samples containing either GST-AtPARP3 or TRXH-AtPARP3. To exclude the possibility that AtPARP3 might need a longer reaction time, we extended the incubation time to 30 min, and no PAR signal was detected (Fig. 1b).

### The AtPARP3 catalytic domain exhibits no activity

**Fig. 2:**
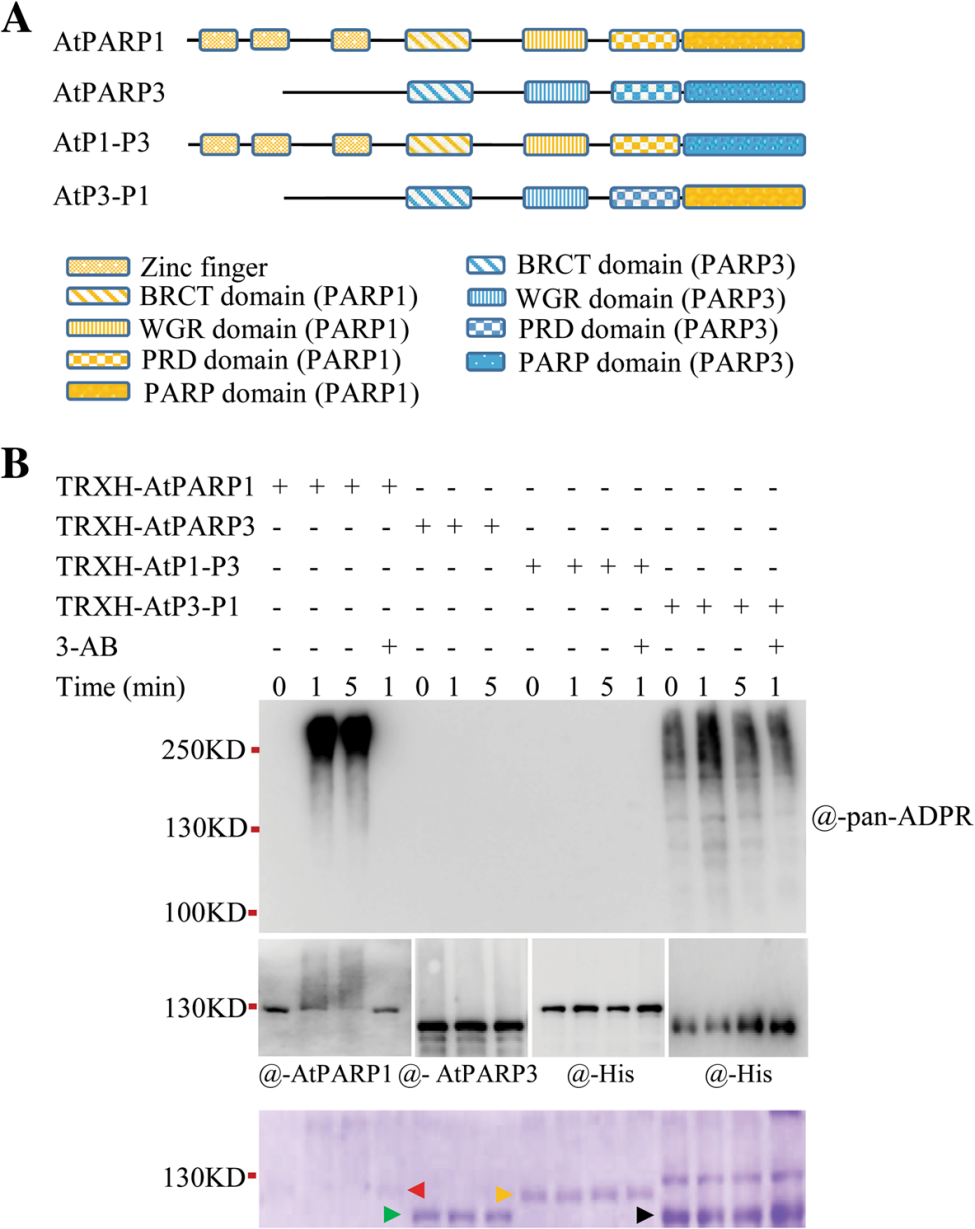
Activity determination of recombinant proteins with domain swapping. **a** Schematic diagram of the domain swapping experiments between AtPARP1 and AtPARP3. Different boxes show different domains. **b** Activity determination of the domain-exchanged proteins. The purified proteins were incubated with 500 nM broken DNA and 1 mM NAD^+^ at 25 °C for different time periods with or without 20 mM 3-AB and then detected by immunoblotting using different antibodies. The arrows in the Coomassie blue-stained SDS-PAGE gel indicate the recombinant proteins TRXH-AtPARP1 (red), TRXH-AtPARP3 (green), TRXH-AtP1-P3 (orange), and TRXH-AtP3-P1 (black). Anti-His antibody was used to detect the expressed TRXH-AtP1-P3 and TRXH-AtP3-P1 proteins

The PARP catalytic domain is important for PARP activity. The N-terminal DNA-binding domain can interact with and activate the C-terminal PARP catalytic domain after binding to DNA [33, 35]. Although AtPARP3 has BRCT, WGR, PRD/HD and PARP catalytic domains, it has no recognized DNA-binding domain in the N-terminal region (Additional file 1: Figure S1); in addition, a C-V-E motif replaces the classical H-Y-E triad in the catalytic core. To assess whether this domain has function in catalyzing PAR formation, we exchanged the catalytic domain of AtPARP1 with that of AtPARP3, as shown in Fig. 2a. The chimera proteins AtP1-P3 and AtP3-P1 were produced in the same expression system as AtPARP1 and AtPARP3. In contrast to the strong activity of AtPARP1, the AtPARP1 protein carrying the AtPARP3 catalytic domain (AtP1-P3) showed no PARP activity (Fig. 2b), which indicated that the AtPARP3 catalytic domain failed to form PAR even in the presence of other functional domains from AtPARP1.

However, the chimera PARP3 protein AtP3-P1, in which most domains of AtPARP3 were retained and only the catalytic domain was replaced by that of AtPARP1 (Fig. 2a), showed constitutive activity, and the PAR signal was detected at all time points, even at the time point “0” in the absence of exogenously supplemented NAD^+^ and DNA (Fig. 2b). 3-AB only slightly reduced the signal, which indicated that the signal had been produced in *E. coli* cells prior to purification*,* and NAD^+^ and DNA supplementation during the assay only mildly enhanced its activity. These results indicated that the catalytic activity of the AtPARP1 domain in AtPARP3 was constitutively “switched on.”

### Other structural elements beyond the catalytic triad also determine activity

**Fig. 3:**
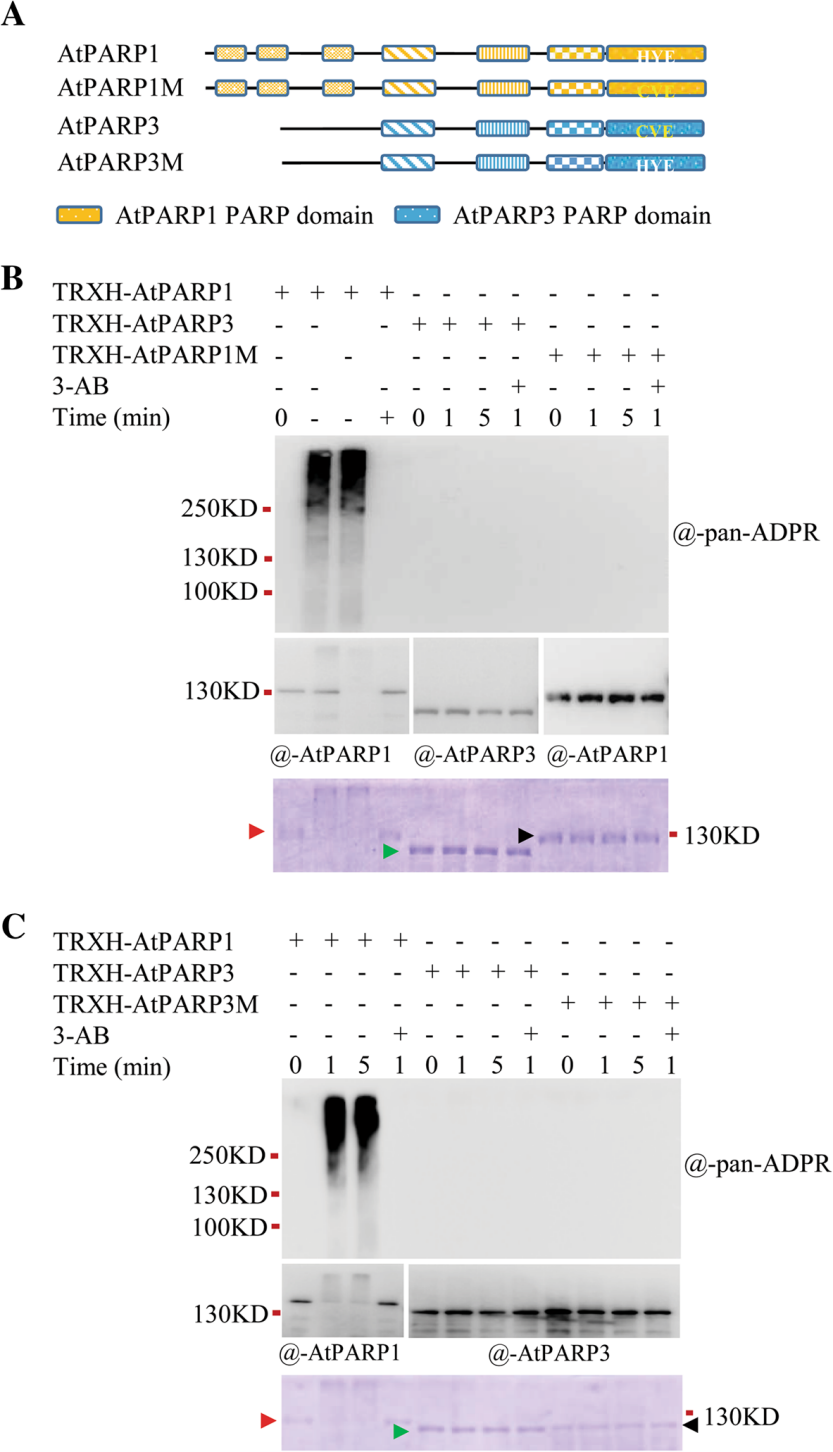
Enzymatic activity of point mutation proteins**. a** Schematic diagrams of the proteins with point mutations. The residues in the catalytic triad are given in the PARP domain. **b** The mutated AtPARP1 protein has no catalytic activity. For TRXH-AtPARP1M, the catalytic triad in AtPARP1 was changed from a normal H-Y-E to C-V-E. **c** Back mutation of the catalytic triad in AtPARP3 cannot recover its activity. For TRXH-AtPARP3M, the catalytic triad in AtPARP3 was changed from C-V-E to a normal H-Y-E. The purified proteins were incubated with 500 nM DNA and 1 mM NAD^+^ at 25 °C for different time periods with or without 20 mM 3-AB. After the reaction, the proteins were analyzed by immunoblot using different antibodies. The arrows in the Coomassie blue-stained SDS-PAGE gel indicate the recombinant proteins TRXH-AtPARP1 (red arrow), TRXH-AtPARP3 (green arrow), TRXH-AtPARP1M or TRXH-AtPARP3M (black arrow)

To understand whether the loss of activity of AtPARP3 is solely due to the change in “H-Y” of the catalytic triad, we mutated the “H-Y” of AtPARP1 to “C-V” (AtPARP1M) by point mutation and changed the “C-V” of AtPARP3 back to “H-Y” (AtPARP3M) (Fig. 3a), and then examined the activities of the AtPARP1 and AtPARP3 mutant proteins as well as those of their wild-type controls. No PAR signal was observed for either the AtPARP1M or AtPARP3M proteins (Fig. 3b and c), which indicated that mutation of the first two amino acids of H-Y-E in AtPARP1 is sufficient to eliminate the PARP enzymatic activity; however, H-Y-E is not the only important motif for determining the activity of AtPARP3 because even a typical H-Y-E triad failed to regenerate the PARP activity in AtPARP3.

Several other motifs are also considered to be important for PARP activity [31], and they varied in AtPARP3. In addition to the catalytic triad motif, AtPARP3 carried altered motifs 1 and 2 in the NAD^+^ fold (Additional file 1: Figure S1B). Histine-glycine-serine (H-G-S) in motif 1 and tyrosine-phenylalanine-alanine (Y-F-A) in motif 2 were replaced by cysteine-glycine-serine (C-G-S) and valine-phenylalanine-alanine (V-C-S), respectively, in AtPARP3, and these two motifs might provide a microenvironment for NAD^+^ binding.

### AtPARP3 loses the ability to bind to NAD^+^

**Fig. 4:**
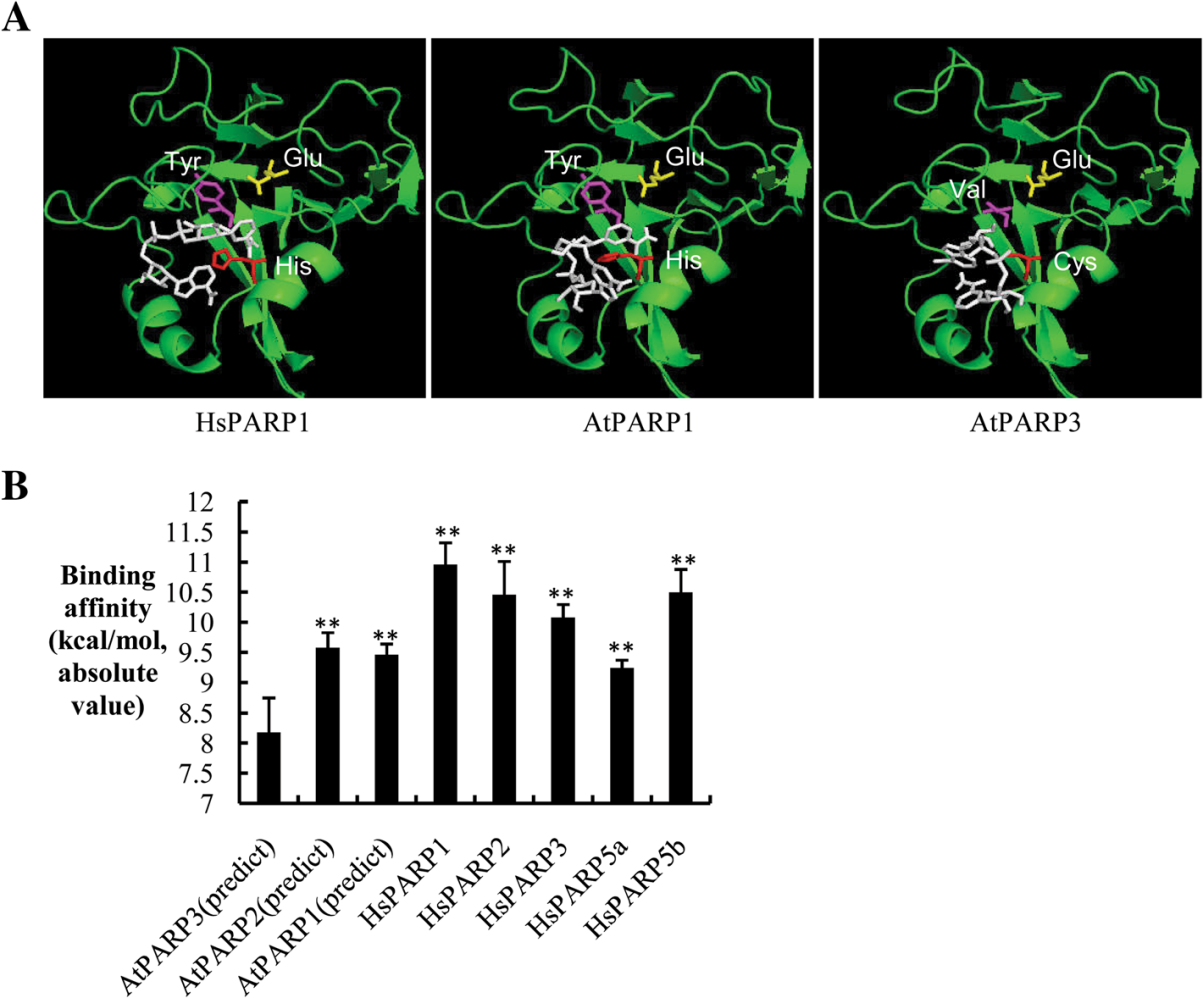
Binding affinity calculations between PARPs and NAD^+^**. a** Molecular docking simulation of PARPs and NAD^+^. Representative images of binding between the PARP catalytic domain (green) and NAD^+^ molecule (white) analyzed using AutoDock software. The histidine (cysteine in AtPARP3), tyrosine (valine in AtPARP3) and glutamate residues of the H-Y-E (C-V-E in AtPARP3) triad motif are labeled in red, magenta and yellow, respectively. The HsPARP1 structure was extracted from a previously published crystal structure (PDB ID: 1UK1). The AtPARP1 and AtPARP3 structures were generated using the homology-based protein structure prediction software Phyre2 [38]. **b** Binding affinities generated by in silico molecular docking. For each protein, the molecular docking experiment was repeated five times with the same parameter settings. The average from the top binding affinity from the various time point was used to evaluate the protein’s binding affinity to the NAD^+^ molecule. The structural information for human HsPARP1, HsPARP2, HsPARP3, HsPARP5a and HsPARP5b was downloaded from PDB, and the structures of *Arabidopsis* PARPs were modeled using Phyre2. Significant differences were determined using Student’s t-test. **P < 0.01

Substrate recognition is the prerequisite for an enzyme to perform activity. To understand the possible structural differences among *Arabidopsis* PARP enzymes, we first simulated the structures of *Arabidopsis* AtPARP1 and AtPARP3 using the resolved crystal structures of HsPARPs as models. Representative images illustrating the binding of the PARP catalytic domain (green) to the NAD^+^ molecule (white) are presented in Fig. 4a. The results indicated that the C and V residues in AtPARP3 failed to correctly orientate the NAD^+^ molecule relative to those of human and *Arabidopsis* PARP1. To investigate whether AtPARP3 retains the capability of binding to NAD^+^, we calculated the binding affinities between PARP proteins and NAD^+^ molecule using AutoDock software [39]. The binding affinity of AtPARP3 to NAD^+^ was significantly lower than those of other known active PARP proteins. Among the PARP proteins, AtPARP1, AtPARP2, HsPARP1 and HsPARP2 were verified as poly (ADP-ribose) polymerases; HsPARP3 is a mono-ADP-transferase; and HsPARP5a and HsPARP5b mediate oligo (ADP-ribosyl) ation [32, 40]. All of these proteins can bind to NAD^+^. The lower binding affinity between AtPARP3 and NAD^+^ implies that AtPARP3 might have lost the capacity to bind to NAD^+^.

**Fig. 5:**
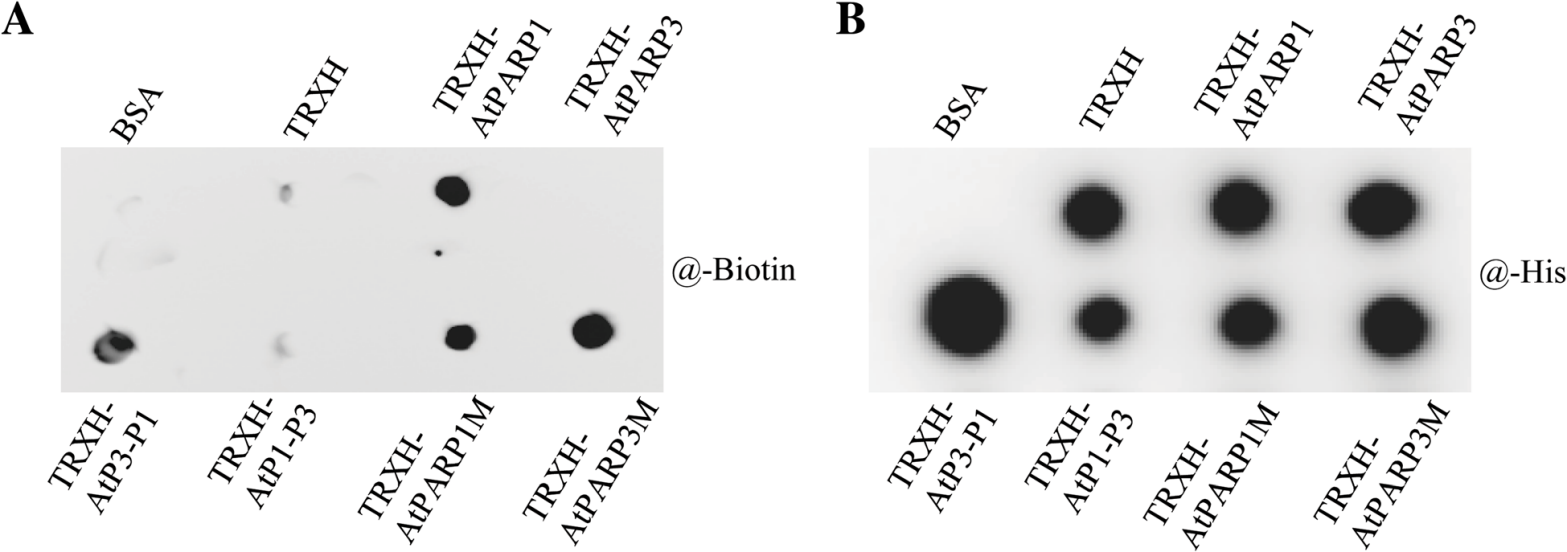
AtPARP3 is incapable of binding to the substrate NAD^+^. **a** Immunoblotting with streptavidin/HRP. **b** Immunoblotting with anti-His antibody. The purified proteins were blotted onto an activated PVDF membrane and then incubated with 25 μM biotinylated NAD^+^ in the presence of 500 nM broken DNA for 30 min at 25 °C. The NAD^+^ binding proteins were detected by blotting with streptavidin/HRP. An anti-His antibody was used to visualize the loading amounts of the proteins blotted on the membrane

To examine the binding activity of NAD^+^ to AtPARP3, we spotted the purified proteins onto a polyvinylidene fluoride (PVDF) membrane, using the irrelevant protein bovine serum albumin (BSA) and the fusion tag protein TRXH as negative controls, and incubated the membrane with biotinylated NAD^+^. If NAD^+^ bound to the proteins on the membrane, the biotin tag on NAD^+^ would allow detection of the NAD^+^ signal by streptavidin/HRP (Fig. 5a). An inner control anti-His antibody was used to visualize the amount of protein spotted on the membrane (Fig. 5b). The results showed that the active enzyme TRXH-AtPARP1 and the chimera TRXH-AtP3-P1 protein were able to bind to NAD^+^, whereas the protein with the AtPARP3 catalytic domains TRXH-AtPARP3 and TRXH-AtP1-P3 could not. Moreover, the inactive protein TRXH-AtPARP3M also showed NAD^+^-binding activity, which suggested that the reverse mutation of C-V-E to H-Y-E recovered the NAD^+^-binding activity of AtPARP3 but failed to recover its PAR-generating activity, and this finding further supported the notion that other motifs around the H-Y-E triad are also important for PARP enzymatic activity. Surprisingly, the AtPARP1 protein with the H-Y-E to C-V-E mutation still maintained NAD^+^-binding activity even though it was inactive, but the underlying molecular basis remains unknown.

### AtPARP3 is not responsible for PAR formation in seeds

PARP modifies itself and other proteins by PARylation; thus, the PAR level in vivo reflects the cellular PARP activity directly. To investigate whether AtPARP3 is physiologically active, comparison of the PAR levels in wild-type plants and loss-of-function mutants of *AtPARP3* is crucial. By examining the PAR levels in *parp3* mutants and in wild-type plants, we could determine whether AtPARP3 is active in vivo.

We ordered T-DNA insertion mutants of *AtPARP3* from TAIR and named them *parp3–1* and *parp3–2*. Quantitative RT-PCR (RT-qPCR) and immunoblotting analyses confirmed that they were both null mutants (Additional file 3: Figure S3A-C). We also included multiple mutants of both *AtPARP1* and *AtPARP2* for comparison. For each gene, at least two different mutants were used, and all these mutants have been used in other studies [4, 20, 22–24, 27, 29]. The T-DNA insertion sites of the mutants for the *AtPARP1* and *AtPARP2* genes are shown in Additional file 3: Figure S3D and E.

**Fig. 6:**
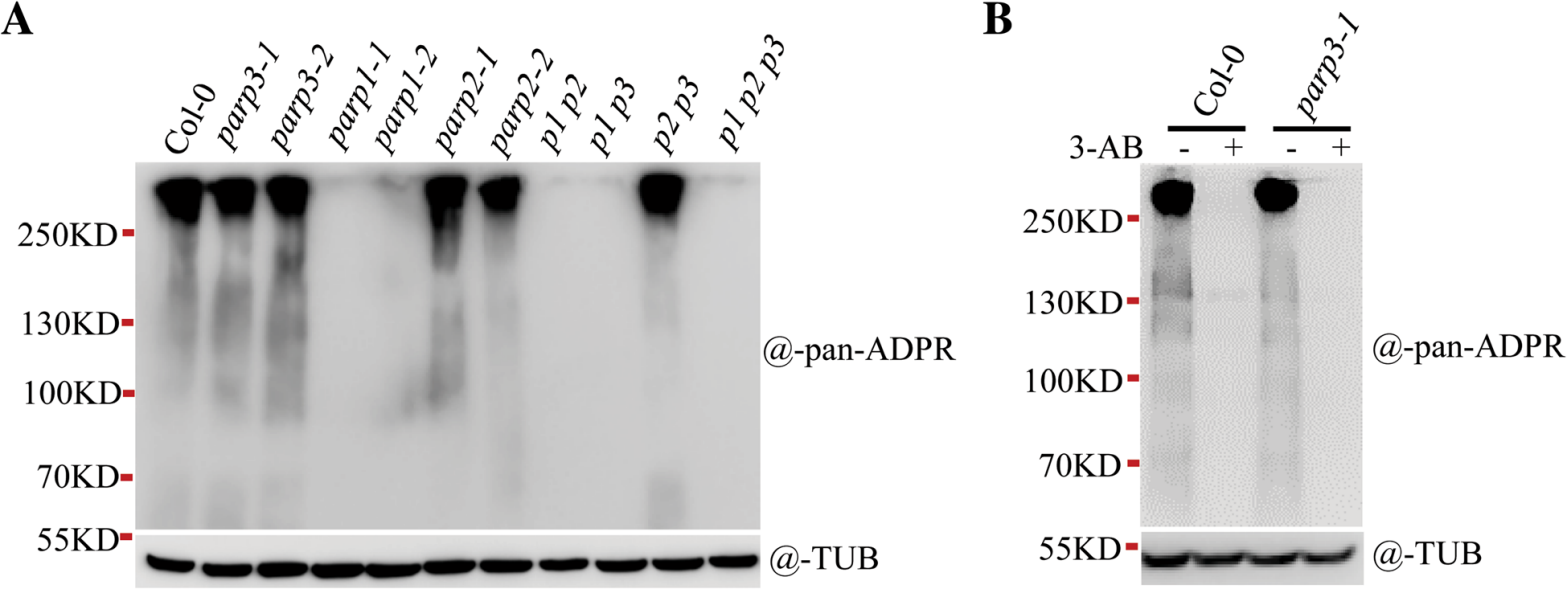
AtPARP3 does not show poly (ADP-ribose) polymerase activity in seeds**. a** PAR signal generated in wild-type and mutant seeds in the presence of broken DNA and NAD^+^. **b** The PARP inhibitor 3-AB can eliminate the PAR signal. Fifty milligrams of wild-type or various mutant seeds was incubated in distilled water for 24 h. Total proteins were extracted from the seeds and then incubated with 1 mM NAD^+^ in the presence of 500 nM broken DNA for 10 min at 25 °C. PAR signals were detected using anti-pan-ADPR reagent. Anti-tubulin antibody was used in the immunoblot assay to show the protein loading amounts. *p1 p2*, *parp1–3 parp2–3*; *p1 p3*, *parp1–1 parp3–1*; *p2 p3*, *parp2–1 parp3–1*; *p1 p2p3*, *parp1–3 parp2–3 parp3–1*; and TUB, tubulin

Because *AtPARP3* is abundantly expressed in seeds but is poorly expressed in other tissues [30] (Additional file 3: Figure S3B and C; Additional file 4: Figure S4A and B), it would be easier to detect its activity in seeds if AtPARP3 had PARP enzymatic activity. AtPARP3 is presumed to play an important role in repairing DNA damage caused by seed dehydration or storage prior to the re-initiation of cell division during germination [30, 31]. As such, it might be an active PARP enzyme in seeds. However, in dry or germinating seeds, no PAR signal could be detected under normal conditions, neither after treatment with genotoxin. The RT-qPCR data also showed that *AtPARP1/2/3* expressions in the early seed germinating stages (within 24 h) were not responsive to zeocin or methyl methanesulfonate (MMS) treatment, which mainly induce double- and single-strand DNA breaks, respectively [41, 42] (Additional file 4: Figure S4C and D). Therefore, we modified our assay to a more robust one. Exogenous NAD^+^ and broken DNA were supplemented in the protein extracts because NAD^+^ is a known rate-limiting factor for PARP activity. In the presence of sufficient substrate (NAD^+^) and activating DNA, any residual PARP activity would be detectable. Surprisingly, we detected PAR formation in the seeds of wild-type, both *parp2* mutants and both *parp3* mutants, but not in the seeds of both *parp1* mutants (Fig. 6a). The PARylated proteins displayed as large smeared bands on the SDS-PAGE gel due to the sufficient provision of NAD^+^ substrate, and the PAR chains were obviously long. The PAR signal strength detected in the two *parp3* mutants was close to that of the wild-type, which indicated that AtPARP3 made no or an undetectable contribution to the PAR signal in seeds. In the *parp1 parp2* (*p1 p2*) double mutant, no PAR signal was detected, which confirmed that AtPARP3 produced no PAR in vivo. Interestingly, in all mutants with the *AtPARP1* mutation, such as the *parp1*, *parp1 parp2*, *parp1 parp3* (*p1 p3*) and *parp1 parp2 parp3* (*p1 p2 p3*) mutants, the PAR signal was undetectable, which indicated that even in seeds, AtPARP1 remains the main PARP responsible for PAR formation, although the expression level of *AtPARP1* in seeds was much lower than that of *AtPARP3* (Additional file 4: Figure S4A). To confirm the validity of the detected PAR signal in seeds, we added the competitive inhibitor 3-AB to the reaction samples. 3-AB eliminated the signal almost completely (Fig. 6b), which indicated that the detected signal was a real PAR signal.

### AtPARP1 has predominant PARP enzymatic activity in response to zeocin and MMS treatments in seedlings

The results obtained in seeds aroused our interest to investigate whether AtPARP1 also plays the predominant PAR formation role in seedlings. In humans, HsPARP1 contributes over 90% of the total PARP activity in vivo and is considered the key member of the PARP family [32, 43]. However, in *Arabidopsis*, AtPARP2, instead of AtPARP1, is reportedly the predominant PARP involved in both DNA damage and biotic stress responses in seedlings [20, 22]. We first examined the expression levels of three *PARP* genes in *Arabidopsis* seedlings and found that *AtPARP1* expression was highest while *AtPARP3* expression was lowest under normal conditions (Additional file 4: Figure S4B). *AtPARP3* could not be induced by zeocin and MMS in both seeds and seedlings (Additional file 4: Figure S4C, D, E, and F), whereas *AtPARP1* and *AtPARP2* transcriptions were strikingly induced by both zeocin and MMS in seedlings (Additional file 4: Figure S4E and F).

**Fig. 7:**
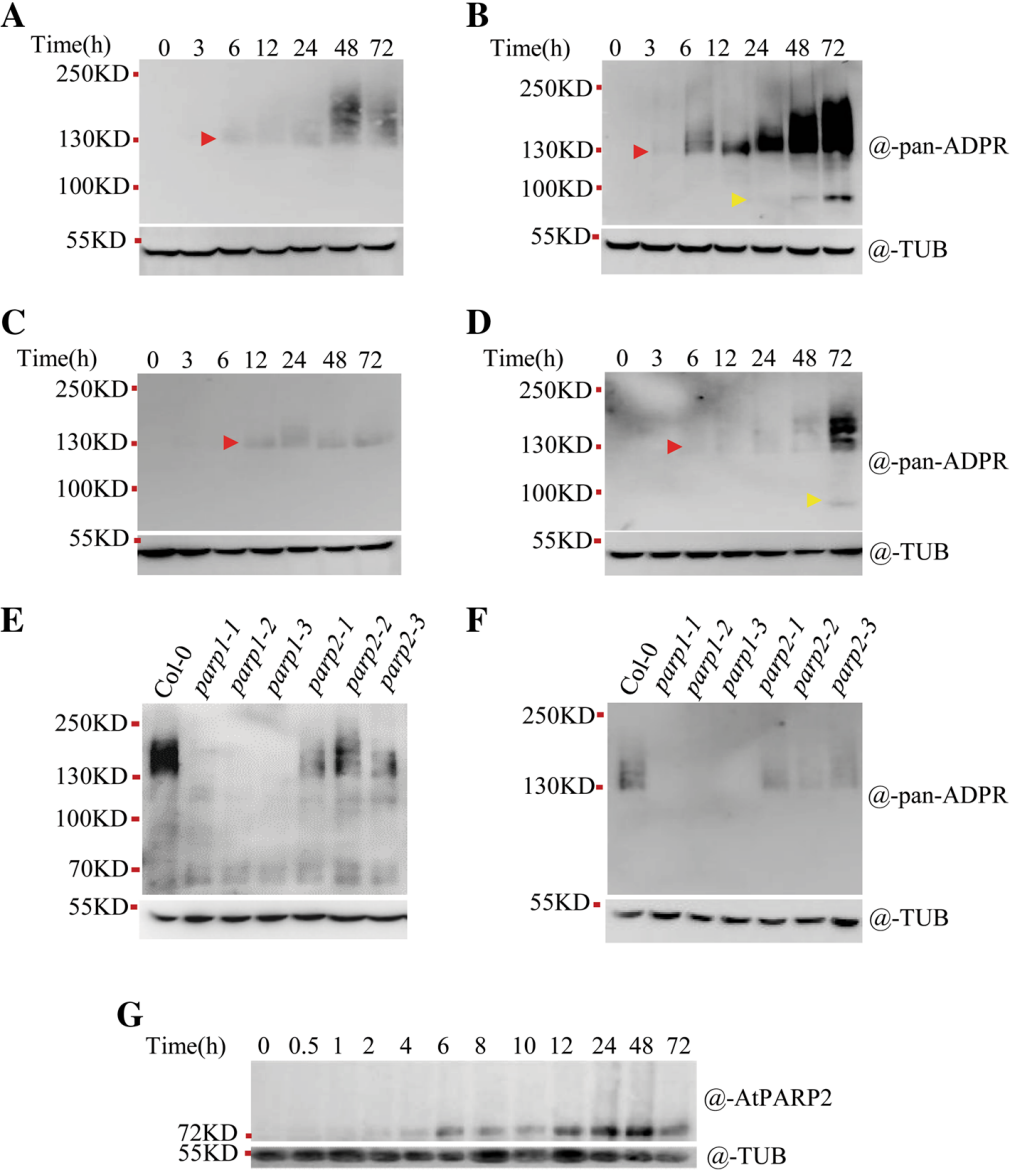
AtPARP1 produces most of the PAR signal under zeocin and MMS treatments. **a** Time-dependent PAR signal generated by wild-type seedlings after zeocin treatment at 100 μg/mL. **b** Time-dependent PAR signal generated by wild-type seedlings after zeocin treatment at 200 μg/mL. **c** Time-dependent PAR signal generated by wild-type seedlings after MMS treatment at 100 μg/mL. **d** Time-dependent PAR signal generated by wild-type seedlings after MMS treatment at 200 μg/mL. **e** PAR signal generated by different mutants of AtPARP1 and AtPARP2 after zeocin treatment at 100 μg/mL for 24 h. **f** PAR signal generated by different mutants of A*tPARP1* and *AtPARP2* after MMS treatment at 200 μg/mL for 24 h. **g** Time-dependent induction of AtPARP2 by zeocin treatment. PAR signal was detected using anti-pan-ADPR reagent. AtPARP2 was detected with an anti-AtPARP2 antibody. Tubulin was detected using an anti-tubulin antibody to show the protein loading amounts. Red arrows indicate the poly (ADP-ribosyl) ated AtPARP1. Yellow arrows indicate the poly (ADP-ribosyl) ated AtPARP2. TUB, tubulin

**Fig. 8:**
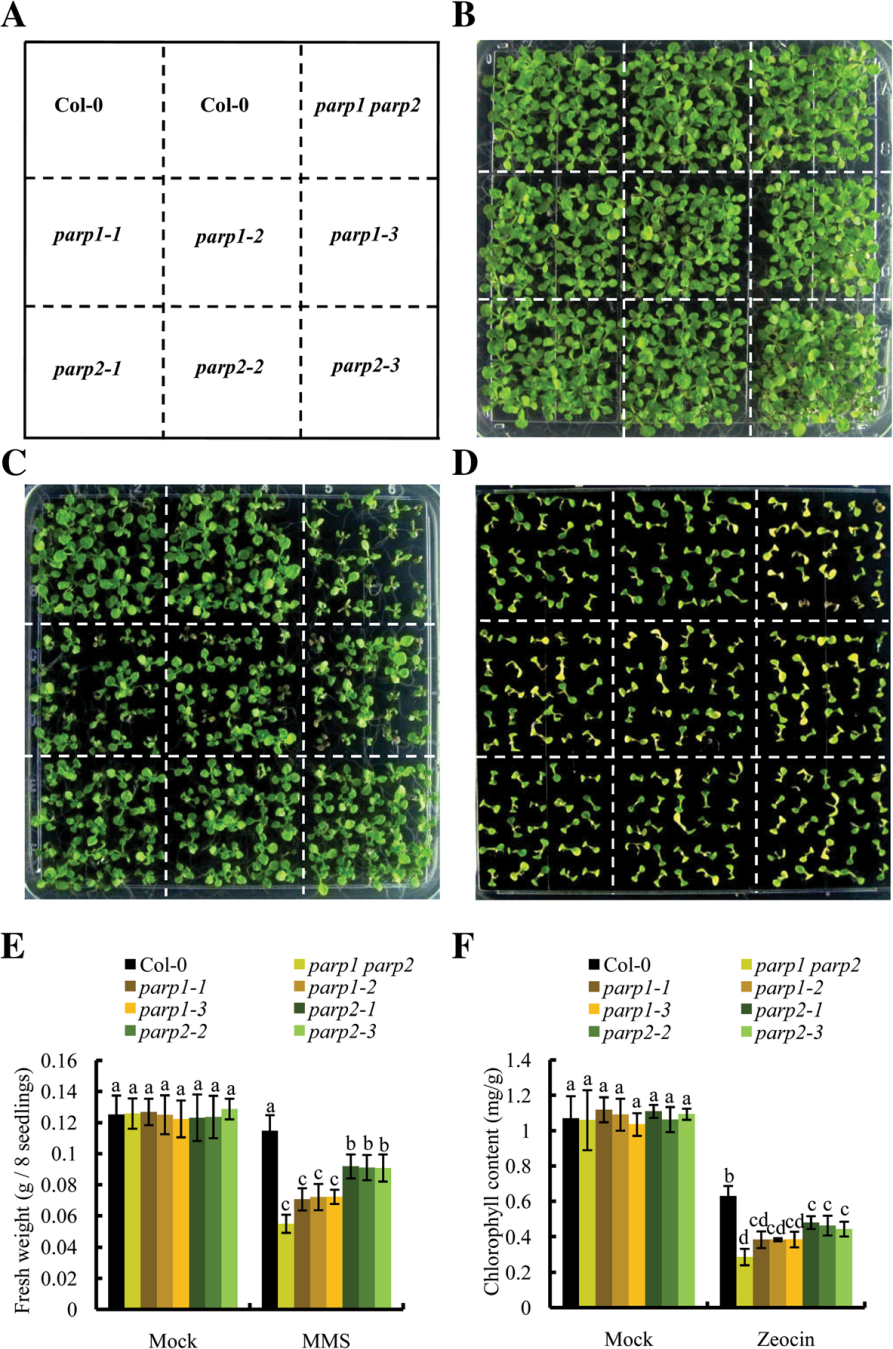
The *parp1* mutants are more sensitive to MMS and zeocin treatments than the *parp2* mutants. **a** Plants arrangement on the plates. **b** Phenotypes of Col-0 and mutants on ½ MS plates without treatment. **c** Phenotypes of Col-0 and mutants on ½ MS plates with 100 μg/mL MMS. **d** Phenotypes of Col-0 and mutants on ½ MS plates with 150 μg/mL zeocin. **e** Fresh weight of per eight seedlings with and without MMS treatment. **f** Chlorophyll content of seedlings with and without zeocin treatment. The seeds were sown on ½ MS plates, stratified for 3 d at 4 °C in the dark, and then transferred into a growth chamber with 16 h of light/8 h of dark. Two-week-old seedlings were used for the assay. Each experiment was repeated at least three times. The results are shown as the means ± SDs from three biological replicates. Two-way ANOVA with Bonferroni post hoc test analysis was performed. Different letters indicate significant differences (*P* < 0.05)

We then used the same *parp* mutants as those used in other laboratories to investigate PAR formation in response to the DNA-damaging agents zeocin and MMS. As expected, PAR was gradually induced in wild-type seedlings after treatment with genotoxin (Fig. 7a-d). All PAR signals originated from physiological PARP activity because the assay was performed without exogenous NAD^+^ and activating DNA. Overall, the PAR signal generated under zeocin treatment was stronger than that under MMS treatment (Fig. 7a-d), which indicated that the stronger double-strand breakage agent zeocin induced more PAR formation than the single-strand break-inducing agent MMS. Longer exposure times and higher concentrations of genotoxins also generated stronger PAR signals. Interestingly, little or no PAR signal was observed in the *parp1–1, parp1–2* and *parp1–3* mutants, and neither did we detect the automodification activity of AtPARP2 in the three *parp1* mutants within 24 h after genotoxin exposure (Fig. 7e and f), whereas AtPARP2 was well induced at these time points in wild-type seedlings (Fig. 7g). Weaker PAR signals were detected in the *parp2–1*, *parp2–2* and *parp2–3* mutants than in Col-0, which indicated that the mutation of AtPARP2 decreased AtPARP1 activity. When grown on plates with MMS, all *parp1* mutants displayed a mildly more sensitive phenotype than the *parp2* mutants (Fig. 8c and e, please see Additional file 5: Table S1 for the source data); while on zeocin plates, although all seedlings could not grow up, the *parp1* mutants had an averagely lower chlorophyll content than the *parp2* mutants (Fig. 8d and f). Taken together, our biochemistry and genetic data support the conclusion that AtPARP1, instead of AtPARP2, makes the greatest contribution to PAR formation in *Arabidopsis* and plays a dominant role in DNA damage response.

### In response to zeocin and MMS treatments, AtPARP1 is first activated, and then AtPARP2 is activated

The time-dependent results of PAR formation in response to DNA-damaging agents (Fig. 7a-d) indicated that a blurred band first appeared around the size of AtPARP1, which is approximately 113 kD. This blurred band disappeared in all *parp1* mutants (Fig. 7e and f), which suggested that it was correlated with AtPARP1. AtPARP1 activation upon genotoxin attack was consistent with the observation in humans that HsPARP1 is rapidly activated by genotoxin [44]. At approximately 48 h after treatment with 200 μg/ml zeocin and 72 h after treatment with 200 μg/ml MMS, a second band, which was the size of AtPARP2 (72 kD), started to appear (Fig. 7b and d), and this band disappeared in the *parp2* mutant (Additional file 6: Figure S5), which indicated that it likely corresponds to AtPARP2. As shown in Fig. 7g, before 48 h AtPARP2 was already highly induced by zeocin (Fig. 7g), but no PAR signal was detected on AtPARP2, which implied that although AtPARP2 was produced, it might not undergo auto-PARylation at this moment.

**Fig. 9:**
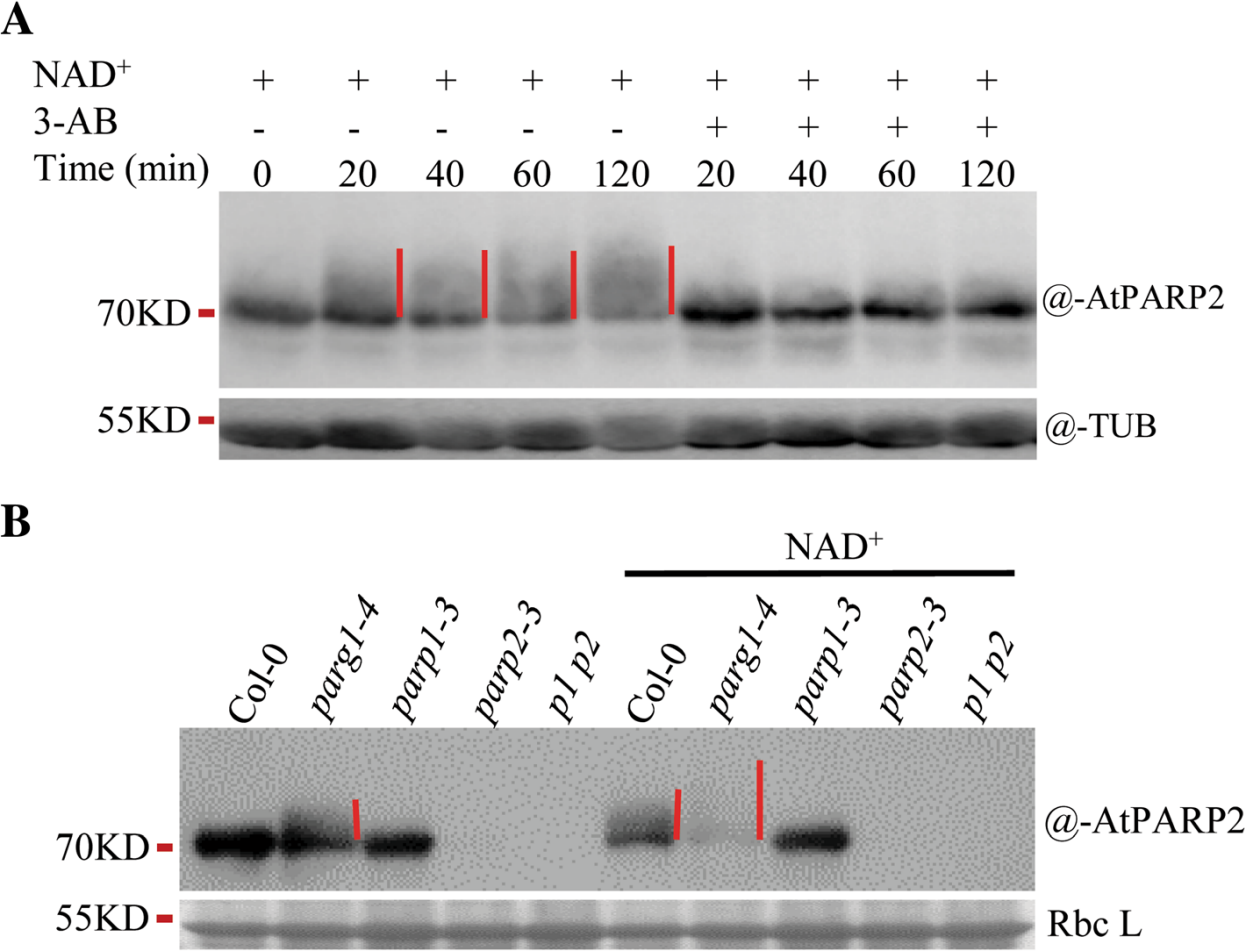
AtPARP2 is poly (ADP-ribosyl) ated by AtPARP1 in vivo**. a** AtPARP2 is poly (ADP-ribosyl) ated in the presence of supplemented NAD^+^ under zeocin treatment. **b** The poly (ADP-ribosyl) ation of AtPARP2 accumulated in the *parg1* mutant and diminished in the *parp1* mutant. The 10-d-old seedlings were treated with 200 μg/mL zeocin for 48 h. The total proteins were extracted with or without exogenously supplemented 1 mM NAD^+^, and PAR signals were detected using anti-pan-ADPR reagent. AtPARP2 was detected with an anti-AtPARP2 antibody. The immunoblot results with anti-tubulin antibody or the Coomassie blue-stained Rubisco large unit (Rbc L) band were used to show the protein loading amounts. Red lines indicate the poly (ADP-ribosyl) ated AtPARP2. TUB, tubulin

To further test whether AtPARP2 was modified by AtPARP1 or by itself in vivo, we examined the changes of the AtPARP2 protein by immunoblot analysis. It was difficult to observe PARylation-induced band shift under physiological conditions when detected by anti-PARP2 antibody, likely due to the negative charges of PAR which weakened the binding of the antibody to the modified proteins, or due to too limited PARylation of AtPARP2 to be observed. To amplify the smear signal typical of PAR formation, we added NAD^+^ to the samples; a very striking upward-blurred band was detected using anti-PARP2 antibody (Fig. 9a), and the upward traces were eliminated by 3-AB, which indicated that AtPARP2 was indeed poly (ADP-ribosyl) ated in the presence of exogenous NAD^+^. To assess whether the catalysis activity originated from AtPARP1 or AtPARP2 itself, we used the *parp1*, *parp2*, *p1 p2* double mutant and *parg1* mutant to observe the homeostasis of the PAR smears. The PAR signal was stronger in the *parg1* mutant because PARG1 is mainly responsible for PAR hydrolysis in vivo, and the mutation of PARG1 allowed the easy detection of PARylated proteins (Fig. 9b). In the *parg1* mutant, we observed very obvious PARylated AtPARP2 at 48 h after zeocin treatment; however, the smearing PAR signal on AtPARP2 was absent in the *parp1* mutant, which indicated that most of the PAR signal on AtPARP2 was generated by AtPARP1, and not by itself. AtPARP2 might not undergo or only undergo very limited auto-PARylation under this condition. This result, together with the previous results that under physiological conditions (without exogenous NAD^+^) the PARylation of AtPARP2 in *parp1* mutants was not detected (Fig. 7 and Additional file 6: Figure S5), suggested that under genotoxic stress, AtPARP1 is first activated and AtPARP2 is subsequently activated, and AtPARP2 is modified by AtPARP1 in vivo.

## Discussion

Defining the biochemical activity of a protein is important for understanding its physiological function. Here, we demonstrated that AtPARP3 has lost PARP enzymatic activity due to the loss of NAD^+^-binding activity, although AtPARP3 is classified into the PARP family based on sequence homology. Therefore, other roles beyond PARP activity should be considered to determine its in vivo function. We also found that AtPARP1 acts as the predominant PARP in *Arabidopsis* in response to zeocin and MMS treatments, and AtPARP2 can be modified by AtPARP1 during the response to DNA damage.

Based on the phylogenetic analysis results (Additional file 2: Figure S2), most of the plant organisms used in this study have only one PARP3 member with the exception of *Glycine max* (two copies), which underwent a recent whole genome duplication event [45]. In addition, in the phylogenetic tree, plant PARP3 clustered close to human PARP1 rather than human PARP3, which suggests that PARP3 might have evolved to serve different functions in plants and animals. Plant PARP3 is mainly expressed in seeds. Because it has no PARP enzymatic activity, AtPARP3 likely plays a novel role, and this finding requires further research.

The crystal structure of human HsPARP1 provides important information for understanding the structure-activity relationship of PARP [33]. This analysis revealed that N-terminal zinc fingers 1 and 3 (Zn1 and Zn3), WGR domain, PRD/HD and the C-terminal catalytic domain (CAT) are the most important domains for PARP activity. Zn1, Zn3 and the WGR domain collectively bind to a double-strand break and cause distortion of the hydrophobic core of PRD/HD, which then reduces the thermal stability of the CAT domain and increases its dynamics [33].

The hydrophobic core of the PRD/HD domain is critical for the regulation of PARP activity. Mutagenesis of the interior leucine residues to hydrophilic amino acids can activate HsPARP1 in the absence of DNA [33], mimicking the DNA damage-induced PRD/HD distortion. We found that a few conserved leucine residues in the PRD/HD domain (from S634 to H768) of AtPARP1 have been replaced by hydrophilic amino acids in the PRD/HD domain of AtPARP3 (from A449 to Y587), which might explain why placement of the AtPARP1 catalytic domain in AtPARP3 (AtP3-P1) results in a constitutively active protein without activating DNA (Fig. 2b). A more hydrophilic core of the PRD/HD domain of AtPARP3 relative to AtPARP1 likely distorted the PRD/HD domain and activated the AtPARP1 catalytic domain in the AtP3-P1 chimera protein. We may create a novel constitutively active PARP protein without the need for DNA activation by utilizing the PRD/HD domain of AtPARP3, which has potential value for studying the functions of PARPs.

Among the 17 members of the human PARP family, only HsPARP1, HsPARP2 and HsPARP3 are known to be involved in DNA repair [2, 32]. These members are evolutionarily closer to each other than to other members. *Arabidopsis* PARP1 and PARP2 correspond to human PARP1 and PARP2, respectively, based on both sequence homology and domain architecture (Additional file 1: Figure S1) [18, 19]. However, *Arabidopsis* PARP2 was previously reported to play the predominant role in both the DNA damage and biotic stress responses [20, 22], and AtPARP2 was shown to have stronger in vitro activity than AtPARP1.

Obviously, our results do not support this conclusion, potentially due to the different experimental conditions used in different studies. Firstly, it is unknown whether the MABE1016 reagent and anti-PAR antibodies have different sensitivities and specificities for different types of PAR. The anti-pan-ADP-ribose binding reagent (MABE1016, Merck), claimed to be able to detect both mono (ADP-ribose) and poly (ADP-ribose), was substantially more sensitive than the anti-poly (ADP-ribose) polymer antibody [10H] (ab14459, Abcam) in our experiments for PAR detection, particularly for the detection of PAR in plant tissues. No signals appeared on the blot when detected by anti-PAR antibody, but strong PAR signals could be observed by anti-pan-ADP-ribose binding reagent using the same samples (Additional file 7: Figure S6A and B). Anti-PAR antibody could only detect the PAR signals when exogenous NAD^+^ and activated DNA were added into the extraction buffer to enhance PARP catalysis reaction (Additional file 7: Figure S6C), but this is not a physiological condition since NAD^+^ amount is limited in vivo. Under this condition, all potential substrates might be PARylated by PARPs. Even so, most of the PAR signals disappeared in three *parp1* mutants, again supporting that AtPARP1 contributes most of the PARP activity.

Secondly, for the in vitro experiments, different protein expression systems were used. Both AtPARP1 and AtPARP3 have 21 cysteine acids, whereas AtPARP2 has only six cysteine acids. Disulfide formation might not be a problem for AtPARP2 but could induce abnormal folding of AtPARP1 in *E. coli*. Therefore, selection of the proper expression vector for AtPARP1 is particularly important. We used the pET32a(+) vector to produce AtPARPs because the thioredoxin tag in this vector can facilitate the correct disulfide bonding of foreign proteins in host strains such as BL21(DE3)trxB^−^ and Origami (DE3), as detailed in the manufacturer’s manual. The recombinant protein TRXH-AtPARP1 showed robust activity in our assays, producing large amounts of PAR within seconds, which is comparable to human PARP1. A maltose-binding domain (MBD) tag was used in previous studies to express recombinant AtPARP1 and AtPARP2 proteins, and in our side-by-side experiments, the MBD-fused AtPARP1 and AtPARP2 were also less active than the TRXH-fused AtPARP1 and AtPARP2, respectively (Additional file 8: Figure S7A and B), and in general, AtPARP2 produced shorter PAR chains than AtPARP1 within the same incubation time periods in our assay.

Finally, for the in vivo assay, different genotoxins have been used to induce DNA breaks. Bleomycin and mitomycin (MMC) treatments were used in a previous study [22], whereas zeocin and MMS were used in our experiments. Although zeocin belongs to the bleomycin family, it exerts milder DNA-damaging effects than bleomycin (product data sheets available at www.calbiochem.com). In our side-by-side experiments with bleomycin and zeocin, we constantly observed that AtPARP1 was responsible for the majority of PAR production (Additional file 9: Figure S8B and C). Under milder genome stimulus, such as treatments with low or medium doses of double- or single-strand DNA-breaking agents, only the activation of AtPARP1 was observed within 2 days (Fig. 7a and c), and after that, AtPARP2 was mainly PARylated by AtPARP1 (Fig. 7, Fig. 9 and Additional file 6: Figure S5). Even so, the possibility that AtPARP2 modifies itself cannot be excluded at the later stages of the DNA damage response or under fiercer DNA-damaging conditions.

Our phenotypic analysis also showed that all three *parp1* mutants were more sensitive to genotoxic stress than the three *parp2* mutants (Fig. 8), supporting an important role of AtPARP1 in the *Arabidopsis* DNA damage response. In animals, AtPARP1 binds to DNA damage sites in less than 1 s [46], and thus, a role in rapid DNA damage detection is assigned to this protein. AtPARP1 might also act as a rapid detector of DNA breaks in plant cells. By interacting with AtPARP2 [22], it modifies AtPARP2 after its self-activation, and then AtPARP1 and AtPARP2 coordinately participate in the DNA repair process. The phylogenetic relationship and the structural similarity strongly indicate that *Arabidopsis* PARP1 is closer to animal PARP1, which contributes more than 90% of PARP activity in vivo [43]. Our biochemical and genetic data also support the notion that AtPARP1, similar to HsPARP1 [46, 47], plays a leading role in the DNA damage response in *Arabidopsis*.

## Conclusions

Taken together, our results demonstrated that AtPARP3 does not act as a PARP enzyme in seeds, which implied that other roles beyond PARP enzymatic activity need to be considered for AtPARP3. In addition, AtPARP1, rather than AtPARP2 and AtPARP3, is the major enzyme responsible for PARylation in both seeds and seedlings in response to zeocin and MMS treatments, supporting a conserved predominant role for PARP1 in the DNA damage response in both animals and plants.

## Methods

### Plant materials and growth conditions

All *Arabidopsis* seeds were of the Col-0 ecotype background. Sequence data of the genes studied in this article can be found on the TAIR website (https://www.arabidopsis.org/) or GenBank database under the following accession numbers: *AtPARP1* (At2g31320), *AtPARP2* (At4g02390), *AtPARP3* (At5g22470), and *AtPARG1* (At2g31870). The Col-0 and T-DNA insertion line seeds *parp3–1* (Salk_108092C), *parp3–2* (Sail_632_D07) and *parg1–4* (Salk_012110) were ordered from the TAIR database. Other T-DNA mutants were kindly provided by the following laboratories: *parp1–1*, *parp1–2*, *parp2–1*, *parp2–2*, *parp1 parp*3, and *parp2 parp3* seeds were obtained from Dr. Edgar Peiter’s Lab at Martin Luther University Halle-Wittenberg, and *parp1–3*, *parp2–3*, and *parp1 parp2* seeds were obtained from Dr. de Pater’s Lab at Leiden University. These lines can be found under the following stock numbers in the TAIR database: *parp1–1* (GK_380E06), *parp1–2* (GK_382F01), *parp1–3* (GK_692A05), *parp2–1* (GK_420G03), *parp2–2* (Sail_1250_B03), and *parp2–3* (Salk_140400).

Homozygous individuals were identified by genomic DNA PCR, RT-qPCR or immunoblotting analysis. The PCR primers used for identification of the mutants are listed in Additional file 10: Table S2. The mutants *parg1–4*, *parp1–1, parp1–2, parp1–3*, *parp2–1, parp2–2, parp2–3*, and *p1 p2* (*parp1–3 parp2–3*) were described previously [23, 24, 29, 30]. *p2 p3* was constructed using *parp2–3 and parp3–1*; *p1 p3* was obtained by crossing *parp1–3* and *parp3–1*; and *p1 p2 p3* was obtained using *parp1–3, parp2–3* and *parp3–1*. The seedlings were grown in a growth chamber under a photoperiodic cycle consisting of 16 h of light and 8 h of dark at 22 °C.

### RNA extraction and RT-qPCR

Total RNA from *Arabidopsis* seedlings was extracted using the TRIzol reagent (TaKaRa, Japan), and the total RNA from seeds was extracted as described previously [48]. RNA was reverse-transcribed with the PrimeScript™ RT reagent kit with gDNA Eraser (TaKaRa, Japan). qPCR was performed with the SYBR Premix Ex Taq™ kit (TaKaRa, Japan) using the CFX96™ Real-Time PCR Detection System (Bio-Rad, USA). The *Arabidopsis UBQ5* gene was used as an internal control to normalize the different samples. The RT-qPCR primers are listed in Additional file 10: Table S2. Three biological replicates were analyzed for each sample.

### Protein expression and ADP-ribosylation assay

The coding sequences of AtPARP1 and AtPARP2 were amplified from the cDNA of 10-d-old seedlings. The *AtPARP1* coding region was cloned into the pET32a(+) vector with the TRXH tag and pMAL-c5G vector with the maltose-binding domain (MBD) tag using *Sac* I/*Not* I and *Nde* I/*Sal* I sites, respectively, and *AtPARP2* was cloned into pET32a(+) using *Bam* HI*/Xho* I sites. The MBP-AtPARP2 plasmid was provided by Dr. He Ping [20]. The AtPARP3 coding sequence was amplified from *Arabidopsis* dry seeds and cloned into the pET32a(+) vector and pGEX-4 T-1 vector with a GST tag using *Sal* I/*Not* I sites. The protein expression constructs were transformed into Origami (DE3) competent cells based on the standard protocol (Novagen, Germany). The empty vectors were also transformed for tag protein expression. The tag proteins served as negative controls in the biochemical activity assay. Recombinant proteins were purified with a column filled with Nickle Sepharose Fast Flow (GE Healthcare, Sweden). The purified proteins were dialyzed completely against 10 mM Tris-HCl (pH 7.5) buffer to remove salts. The proteins (1–10 μg) were aliquoted into each tube containing 20 mM Tris-HCl (pH 7.5), 50 mM NaCl, 7.5 mM MgCl_2_, 0.2 mM DTT, and 500 nM synthesized DNA oligos [35]. To initiate the reaction, NAD^+^ (Sigma Aldrich, USA) was added to each tube to a final concentration of 1 mM. The reactions were continued at room temperature for the desired time periods and then terminated by adding a 3-fold volume of prechilled acetone. After centrifugation, the precipitated proteins were subjected to SDS-PAGE for immunoblotting assays or Coomassie blue staining. The immunoblots were probed with anti-pan-ADP-ribose binding reagent (Merck, Germany), anti-His antibody (Proteintech, USA), anti-AtPARP1 polyclonal antibody (against the *E.coli*-expressed full-length AtPARP1) (Youke Biotech, China) and anti-AtPARP3 polyclonal antibody (against the *E.coli*-expressed AtPARP3 fragment 160S-392K) (Youke Biotech, China), respectively for detecting different signals. After chemiluminescence reaction, the protein bands were visualized using a ChemScope 3300 mini instrument (CLINX, China).

### Dot blot

The purified proteins were blotted onto the methanol-activated PVDF membrane (Millipore, USA). After drying at room temperature, the membranes were incubated with 25 μM biotinylated NAD^+^ (Trevigen, USA) and 500 nM synthesized DNA oligos for 30 min at 25 °C. The blot was washed with TBST buffer (50 mM Tris, 0.5 M NaCl, and 0.05% Tween-20, pH 8.0) and then incubated with streptavidin/HRP (Solarbio, China) at room temperature for 2 h. After reaction with chemiluminescent substrates, fluorescence signals were detected using a ChemScope 3300 mini instrument (CLINX, China). BSA and tag proteins were used as negative controls, and AtPARP1 was used as the positive control. The signals obtained with anti-His antibody (Proteintech, USA) were used as the protein loading control for each spot.

### Protein extraction and immunoblotting

To detect the PAR signal in seedlings, 10-d-old seedlings were treated with different concentrations of zeocin or MMS for 48 h. The seedlings (100 mg) were homogenized in liquid nitrogen and then suspended in protein extraction buffer (50 mM Tris-HCl pH 8.0, 100 mM NaCl, 5 mM MgCl_2_, 10% glycerol, 0.1% NP-40 and protease inhibitor cocktail). The supernatant was isolated by centrifugation, and after SDS loading buffer was added to the sample, the sample tube was boiled for 10 min. After SDS-PAGE analysis, the proteins were blotted onto Immobilon-P Transfer membranes (Millipore, USA). The PAR levels were detected using anti-Pan-ADPR binding reagent or anti-poly (ADP-ribose) polymer antibody [10H] (ab14459, Abcam, UK). AtPARP2 was detected using an anti-AtPARP2 polyclonal antibody (against the *E.coli*-expressed full-length AtPARP2) (Youke Biotech, China), and tubulin was detected with anti-tubulin antibody (Beyotime, China) as a loading control. The protein signals were visualized using a ChemScope 3300 mini instrument (CLINX, China).

To detect the PAR signal in seeds, dry seeds (50 mg) were imbibed in distilled water for 24 h, and the total proteins were extracted using protein extraction buffer. After carefully removing the surface-floating insoluble and fatty acid components, the supernatant proteins were incubated for 10 min at 25 °C with 1 mM NAD^+^ and 500 nM DNA oligos. The immunoblotting was performed as described above.

### Molecular docking and binding affinity calculation

The protein structures of HsPARP1, HsPARP2, HsPARP3, HsPARP5a and HsPARP5b have been resolved, and the structural data can be downloaded from the RCSB protein data bank (PDB, https://www.rcsb.org/). No crystal structures are available for AtPARP1, AtPARP2 and AtPARP3; therefore, we used the online structure prediction software Phyre2 [38] (http://www.sbg.bio.ic.ac.uk/phyre2/html/page.cgi?id=index) to predict their structures. The generated structures of AtPARP1, AtPARP2 and AtPARP3 together with the resolved structures of HsPARP1, HsPARP2, HsPARP3, HsPARP5a and HsPARP5b were used for molecular docking.

The structure file for NAD^+^ was generated using ChemBio3D software. The open source program AutoDock Vina [39] was used to perform the molecular docking experiment. Specifically, the protein was set as a rigid structure, and the NAD^+^ molecule was flexible, with all of the rotatable bonds (17 out of 32) free to rotate. The coordinates of the alpha carbon in histidine (cysteine in AtPARP3) were used to center the docking grid box because the histidine and tyrosine residues of the triad motif are known to be involved in NAD^+^ binding. The size of the docking grid box was 22.5∗22.5∗22.5 (in Angstrom units) for all proteins.

For each protein, the molecular docking experiment was repeated five times with the same parameters, and nine binding modes with different binding affinities were generated each time. The mean of the top binding affinity from the various time points was used to evaluate the protein’s binding affinity to the NAD^+^ molecule.

### Sequence and motif analyses

Alignments of the PARP protein sequences were performed using the MAFFT server (https://www.ebi.ac.uk/Tools/msa/mafft/) [49]. The conserved functional domains of PARPs were predicted with the NCBI CDD tool (www.ncbi.nlm.nih.gov/cdd) using the structural information provided by the TAIR server (www.*Arabidopsis*.org) as a reference.

### Identification of PARP1/2/3 subfamily members and phylogenetic analysis

Twenty-eight representative species were selected for the phylogenetic analysis of the PARP1/2/3 subfamily in the PARP gene family (Additional file 11: Table S3). The genomic and proteomic sequences of 19 selected species (*Amborella trichopoda, Arabidopsis thaliana, Brassica rapa, Capsella rubella, Carica papaya, Cucumis sativus, Glycine max, Medicago truncatula, Mimulus guttatus, Oryza sativa, Phaseolus vulgaris, Physcomitrella patens, Populus trichocarpa, Theobroma cacao, Selaginella moellendorffii, Sorghum bicolor, Solanum tuberosum, Vitis vinifera* and *Zostera marina*) were downloaded from Phytozome version 12 [50]. The proteomic sequences of *Nelumbo nucifera, Picea abies* and *Ginkgo biloba* were downloaded from https://omictools.com/lotus-db-tool [51], ConGenIE [52] and GigaDB [53], respectively. The proteomic sequences of four animal (*Caenorhabditis elegans, Drosophila melanogaster, Homo sapiens,* and *Mimulus guttatus*) and two fungal (*Mycena alexandri* and *Sarcoscypha coccinea*) species were retrieved from Ensembl [54] and MycoCosm [55], respectively.

To identify the members of the PARP1/2/3 subfamily, the protein sequences of AtPARP1, 2 and 3 were used as queries to blast against the proteomes of the 28 selected species using DIAMOND blastp [56] with E-value ≤1 × 10^− 5^. According to the Pfam analysis [57], the target dataset was screened to remove sequences without a WGR, PARP_reg or PARP domain. Only homologs containing all three domains with identity > 50 were selected as PARP1/2/3 subfamily proteins in this study. Multiple protein sequences were aligned using MAFFT [49] with the default parameters, manually adjusted using AliView [58], and then trimmed using trimAL [59] with the option “-gt 0.6”. To illustrate the evolutionary relationships between the PARP1/2/3 subfamilies, the maximum likelihood (ML) phylogenetic tree was constructed using RAxML version 8.1.17 [60] with the “PROTCATJTT” model. The bootstrap significance test was performed with 100 replicates.

### Chlorophyll extraction

Approximately 20 mg of total seedlings was homogenized in liquid nitrogen and then extracted in 95% acetone for 15 min in the dark. The supernatant solution was isolated by centrifugation for 10 min and used to determine the OD645 and OD663 values through a spectrophotometric analysis. The total chlorophyll content was calculated according to the following formula: Chl a + Chl b = (20.21 ∗ OD645 + 8.02 ∗ OD663) / 1000 ∗ volume / weight.

### Domain swapping and point mutagenesis of PARP proteins

The recombinant protein AtP1-P3 includes amino acids 1–628 of AtPARP1 and amino acids 424–814 of AtPARP3. The AtP3-P1 protein consists of amino acids 1–423 of AtPARP3 and amino acids 629–984 of AtPARP1. The cDNA fragments of these regions were ligated using a NovoRec® PCR one-step clone kit (SinoBio, China). For point mutations, the catalytic triad of AtPARP1 is composed of three amino acids, H833-Y867-E960, whereas that of AtPARP3 consists of the C653-V687-E782 amino acids. The coding sequences of proteins with the catalytic triad point mutations were obtained by a two-step PCR site-directed method [61]. In detail, the DNA sequence of AtPARP1 or AtPARP3 was divided into two fragments. The first point mutation was designed in one pair of primers, and PCR was performed to amplify the first fragment. The second point mutation was then included in another pair of primers and accomplished using the same method. The two PCR fragments were ligated by PCR using the full-length primers. The full-length fragment with two point mutations was cloned into the pET32a(+) vector using *Sal* I/*Not* I sites. All the primers used in the above experiments are listed in Additional file 10: Table S2.

## Additional files

Additional file 1: **Figure S1** Domain architecture and sequence analysis of PARP members in *Arabidopsis* and humans. (A) Comparison of the domain architecture between human and *Arabidopsis* PARP1, PARP2 and PARP3. (B) Motif-based sequence alignment of the PARP signature of *Arabidopsis* PARP1, PARP2 and PARP3. Stars show the conserved H-Y-E triad in AtPARP1 and AtPARP2, which has an alternate form in AtPARP3. Red frames show the sequence motifs 1, 2 and 3. The letters with the same color represent conserved amino acids. (PDF 10681 kb)

Additional file 2: **Figure S2** Maximum likelihood (ML) phylogenetic tree of the PARP1/2/3 subfamily members in 28 representative species. The *Arabidopsis thaliana* PARP3 protein is marked by a red solid triangle. Orange solid asterisks on nodes denote gene duplication events. The bootstrap values (> 50) with 100 replicates are given for each node on the tree. Genes from plants, animals and fungi are given in Additional file 11: Table S3. (PDF 8823 kb)

Additional file 3: **Figure S3** Mutants of *Arabidopsis PARP* genes used in this study. (A) T-DNA insertion sites in *Arabidopsis parp3* mutants. *parp3–1*, Salk_108092C; *parp3–2*, Sail_632_D07. Exons are represented by filled boxes, introns are represented by dark lines, and T-DNA insertions are indicated by filled triangles. (B) RT-qPCR analysis of the *AtPARP3* expression levels in Col-0 and *parp3* mutant seeds. Dry seeds were used for RT-qPCR. The *AtUBQ5* gene was used as the internal control. (C) Detection of AtPARP3 protein in seeds with anti-AtPARP3 antibody. Fifty milligrams of dry seeds was used for the extraction of total protein from each sample. The blotting results with anti-tubulin antibody served as loading controls. (D) T-DNA insertion sites in *parp1* mutants. *parp1–1*, GK_380E06; *parp1–2*, GK_382F01; and *parp1–3*, GK_692A05. (E) T-DNA insertion sites in *parp2* mutants. *parp2–1*, GK_420G03; *parp2–2*, Sail_1250_B03; and *parp2–3*, Salk_140400. The results are shown as the means ± SDs from three biological replicates. TUB, tubulin. (PDF 4047 kb)

Additional file 4: **Figure S4** Transcription levels of *AtPARPs* under normal conditions and genotoxin treatments in seeds and seedlings. (A) Relative expression levels of the *AtPARP* members in *Arabidopsis* dry seeds. (B) Expression levels of the *AtPARP* members in *Arabidopsis* seedlings after distilled water treatment. (C) Comparison of the expression levels of the *AtPARP* members in seeds after MMS treatment. (D) Comparison of the expression levels of the *AtPARP* members in seeds after zeocin treatment. (E) Comparison of the expression levels of the *AtPARP* members in *Arabidopsis* seedlings after MMS treatment. (F) Comparison of the expression level of the *PARP* members in *Arabidopsis* seedlings after zeocin treatment. Seeds of Col-0 and *parp* mutants were incubated with distilled water (A), 100 μg/mL MMS (C), or 200 μg/mL zeocin (D) for different time periods. 10-d-old Col-0 and *parp* mutants seedlings grown on ½ MS plates were sprayed with distilled water (B), 100 μg/mL MMS (E), or 200 μg/mL zeocin (F) for different time periods. Total RNA was extracted from seeds or seedlings and subjected to RT-qPCR analysis. The expression levels of *AtPARPs* were normalized to that of *AtUBQ5*. Simultaneous mock experiments were performed by treating plants with distilled water, and fold changes were calculated by normalizing the gene expression levels under treatment to that of the corresponding mock experiment. The results are shown as the means ± SDs from three biological replicates. (PDF 12025 kb)

Additional file 5: **Table S1** Source data of seedling fresh weight and chlorophyll content under genotoxin treatment. (XLSX 32 kb)

Additional file 6: **Figure S5** Comparison of PAR signals in Col-0, *parp1* and *parp2* mutants. 10-d-old seedlings were treated by 200 μg/mL zeocin for 48 h. The total proteins in the seedlings were extracted, blotted and then detected using anti-pan-ADPR reagent. Tubulin was detected using an anti-tubulin antibody to show the protein loading amounts. TUB, tubulin. (PDF 2355 kb)

Additional file 7: **Figure S6** Comparison of the PAR signals detected by anti-pan-ADPR reagent and anti-PAR antibody, respectively. **(A)** PAR signals in different plants were detected by anti-pan-ADP-ribose binding reagent. **(B)** PAR signals in different plants were detected by anti-PAR antibody. No signal could be detected on the membrane. **(C)** PAR signals in different plants were detected by anti-PAR antibody with exogenous NAD^+^ and activated DNA in the extraction buffer. 0.3 mM NAD^+^ and 100 nM broken DNA were added into the protein extraction buffer to enhance the PARP catalysis reactions. For (A), (B) and (C), 10-d-old seedlings were treated by 200 μg/mL zeocin for 48 h and the total proteins in the seedlings were extracted and used for western blot. For (B), the samples are the same as those in (A) but detected with anti-PAR antibody. (C), Total proteins were extracted using the same buffer as (A) and (B) except in it 0.3 mM NAD^+^ and 100 nM broken DNA were added. TUB, tubulin; @-pan-ADPR, anti-pan-ADPR reagent; @-PAR, anti-PAR antibody. (PDF 7691 kb)

Additional file 8: **Figure S7** Comparison of the activities of MBD-fused and TRXH-fused recombinant AtPARP proteins. (A) Comparison of the activities of different tag-fused AtPARP1 proteins. (B) Comparison of the activities of different tag-fused AtPARP2 proteins. The purified proteins were incubated with 500 nM DNA and 1 mM NAD^+^ at 25 °C for different time periods. After the reaction, the proteins were analyzed by immunoblotting with anti-pan-ADPR reagent (the upper panel). Arrows in the bottom Coomassie blue-stained gel indicate the recombinant proteins TRXH-AtPARP1 (red), MBD-AtPARP1 (black), TRXH-AtPARP2 (cyan), and MBD-AtPARP2 (blue). (PDF 7207 kb)

Additional file 9: **Figure S8** AtPARP1 was responsible for the generation of most of the PAR signals under bleomycin and zeocin treatments. **(A)** PAR signals in different *parp1* and *parp2* mutants after mock (H_2_O) treatment for 24 h and 48 h, respectively. **(B)** PAR signals in different *parp1* and *parp2* mutants after 200 μg/mL zeocin or 25 μg/mL bleomycin treatment for 24 h. **(C)** PAR signals in different *parp1* and *parp2* mutants after 200 μg/mL zeocin or 25 μg/mL bleomycin treatment for 48 h. 10-d-old seedlings were treated with zeocin or bleomycin for different time periods and then the total proteins were extracted and detected using anti-pan-ADPR reagent. Tubulin was detected using an anti-tubulin antibody to indicate the protein loading amounts. TUB, tubulin; @-pan-ADPR, anti-pan-ADPR reagent. (PDF 9141 kb)

Additional file 10: **Table S2** Primers used in this work. (PDF 12294 kb)

Additional file 11: **Table S3** List of genes used for phylogenetic analysis of the PARP1/2/3 subfamily in the PARP family. (PDF 10506 kb)

## Data Availability

The datasets supporting the conclusions described in this article are included within the manuscript and its additional files, and the raw data are available from the corresponding author upon reasonable request.

## Abbreviations

3-AB: 3-aminobenzamide
BRCT: BRCA-1 C-terminus
BSA: albumin from bovine serum
CAT: C-terminal catalytic domain
C-G-S: cysteine-glycine-serine
C-V-E: histidine-valine-glutamic acid
GST: glutathione S-transferase
HD: Helical subdomain
H-G-S: histine-glycine-serine
H-Y-E: histidine-tyrosine-glutamic acid
MAMP: microbe- associated molecular pattern
mART: mono (ADP-ribosyl)ation
MBD: maltose-binding domain
MMC: mitomycin
MMS: methyl methanesulfonate
PAR: poly (ADP-ribose)
PARG: poly (ADP-ribose) glycohydrolase
PARP: poly (ADP-ribose) polymerase
PARylation: poly (ADP- ribosyl)ation
PRD: PARP regulatory domain
PVDF: polyvinylidene fluoride
SAP: SAF/Acinus/PIAS motif
TRXH: thioredoxin and histidine
V-C-S: valine-phenylalanine-alanine
WGR: Trp-Gly-Arg
Y-F-A: tyrosine-phenylalanine-alanine
