# Supplementary figures and images for: New perspectives on the plant PARP family: Arabidopsis PARP3 is inactive, and PARP1 exhibits predominant poly (ADP-ribose) polymerase activity in response to DNA damage
Source: BMC Plant Biol. 2019 Aug 19;19:364. doi: 10.1186/s12870-019-1958-9 (PMC6701155; doi:10.1186/s12870-019-1958-9)

(A)

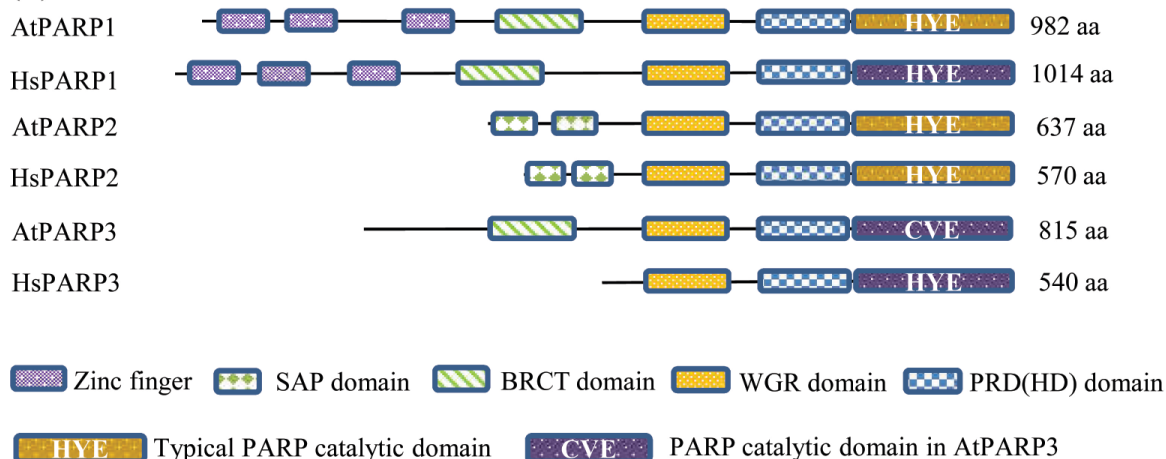

(B)

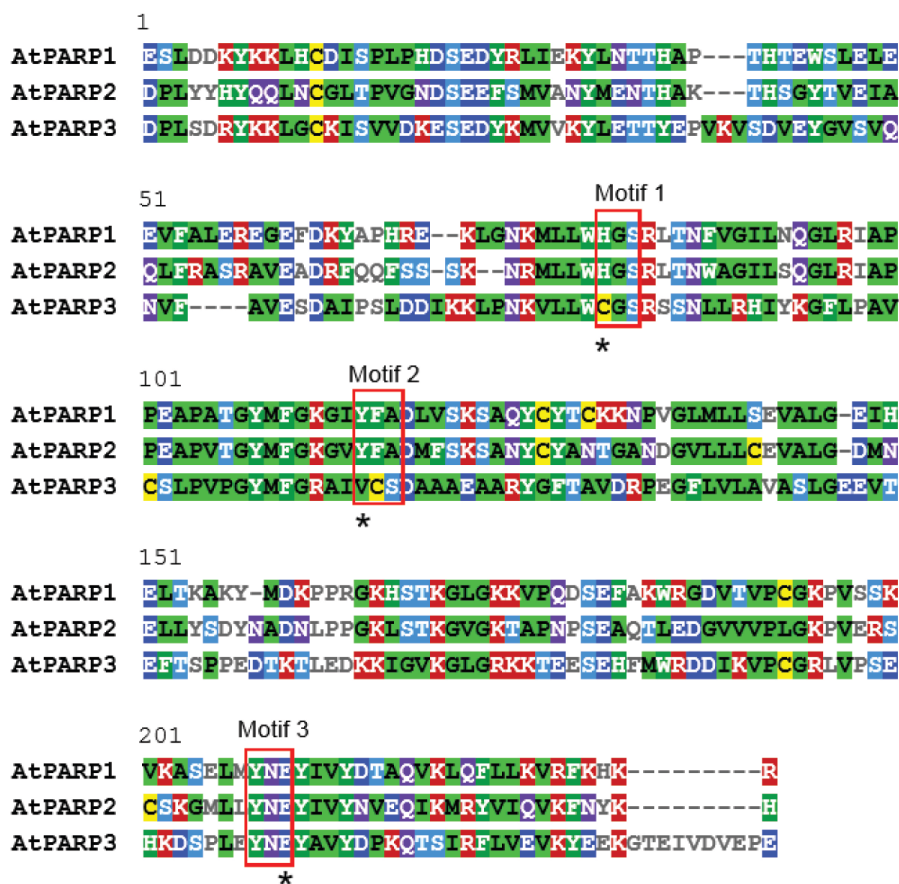

Supplement: Supplementary file 1 — Figure S1 Domain architecture and sequence analysis of PARP members in Arabidopsis and humans. (A) Comparison of the domain architecture between human and Arabidopsis PARP1, PARP2 and PARP3. (B) Motif-based sequence alignment of the PARP signature of Arabidopsis PARP1, PARP2 and PARP3. Stars show the conserved H-Y-E triad in AtPARP1 and AtPARP2, which has an alternate form in AtPARP3. Red frames show the sequence motifs 1, 2 and 3. The letters with the same color represent conserved amino acids. (PDF 10681 kb) [file 12870_2019_1958_MOESM1_ESM.pdf]

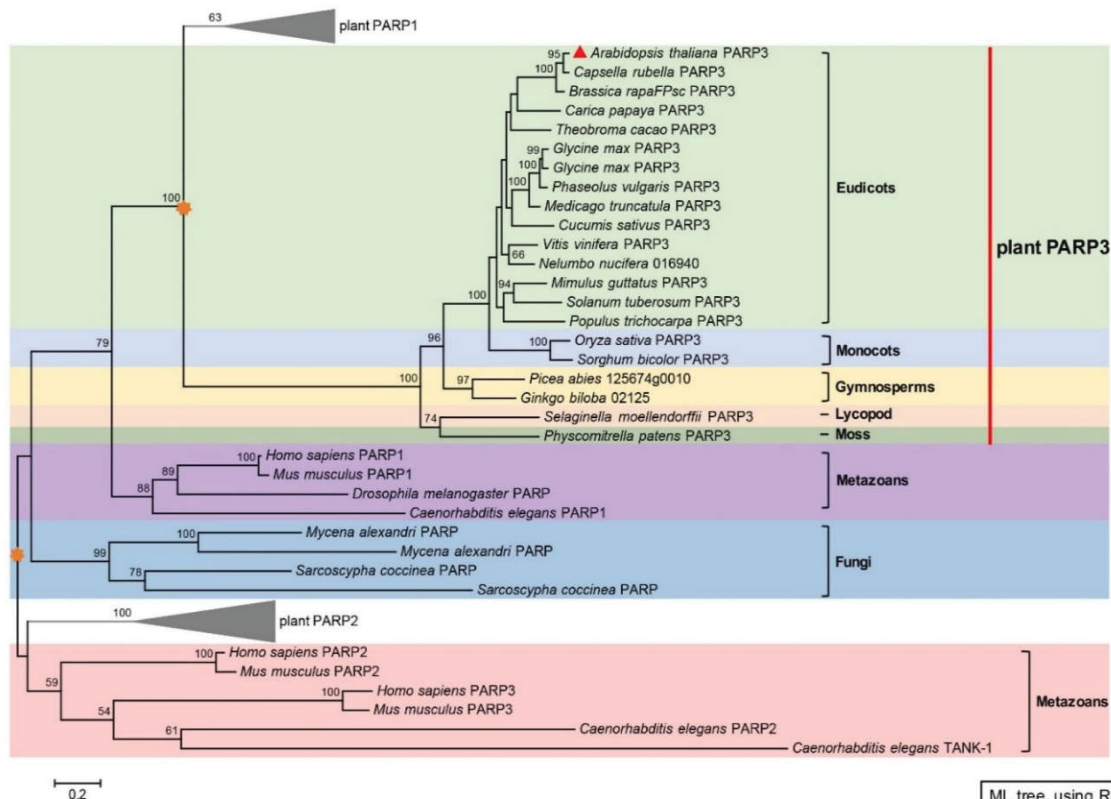

Supplement: Supplementary file 2 — Figure S2 Maximum likelihood (ML) phylogenetic tree of the PARP1/2/3 subfamily members in 28 representative species. The Arabidopsis thaliana PARP3 protein is marked by a red solid triangle. Orange solid asterisks on nodes denote gene duplication events. The bootstrap values (> 50) with 100 replicates are given for each node on the tree. Genes from plants, animals and fungi are given in Additional file 11: Table S3. (PDF 8823 kb) [file 12870_2019_1958_MOESM2_ESM.pdf]

(A)

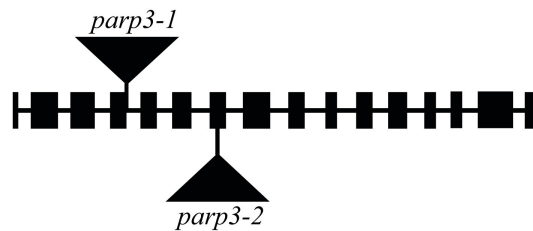

(B)

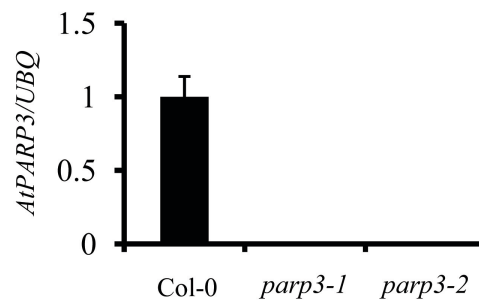

(C)

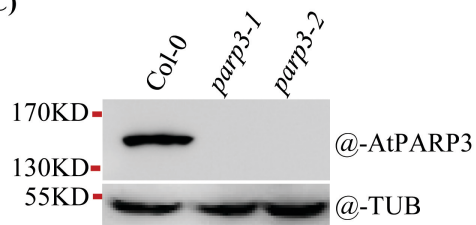

(D)

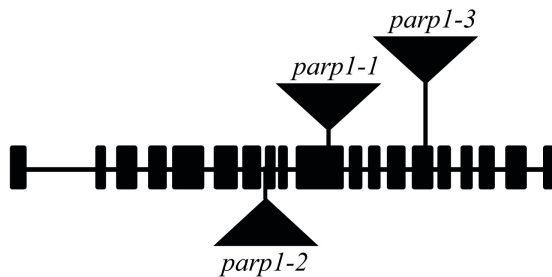

(E)

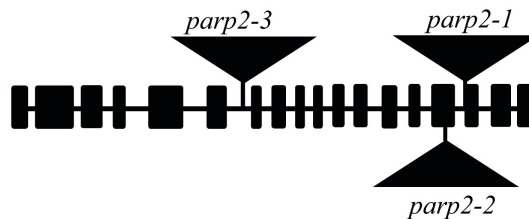

Supplement: Supplementary file 3 — Figure S3 Mutants of Arabidopsis PARP genes used in this study. (A) T-DNA insertion sites in Arabidopsis parp3 mutants. parp3–1, Salk_108092C; parp3–2, Sail_632_D07. Exons are represented by filled boxes, introns are represented by dark lines, and T-DNA insertions are indicated by filled triangles. (B) RT-qPCR analysis of the AtPARP3 expression levels in Col-0 and parp3 mutant seeds. Dry seeds were used for RT-qPCR. The AtUBQ5 gene was used as the internal control. (C) Detection of AtPARP3 protein in seeds with anti-AtPARP3 antibody. Fifty milligrams of dry seeds was used for the extraction of total protein from each sample. The blotting results with anti-tubulin antibody served as loading controls. (D) T-DNA insertion sites in parp1 mutants. parp1–1, GK_380E06; parp1–2, GK_382F01; and parp1–3, GK_692A05. (E) T-DNA insertion sites in parp2 mutants. parp2–1, GK_420G03; parp2–2, Sail_1250_B03; and parp2–3, Salk_140400. The results are shown as the means ± SDs from three biological replicates. TUB, tubulin. (PDF 4047 kb) [file 12870_2019_1958_MOESM3_ESM.pdf]

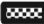 *AtPARP1*
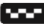 *AtPARP2*
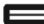 *AtPARP3*

(A)

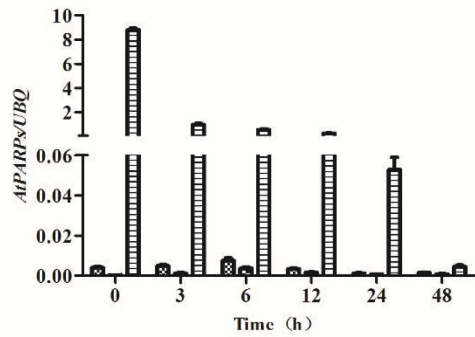

(B)

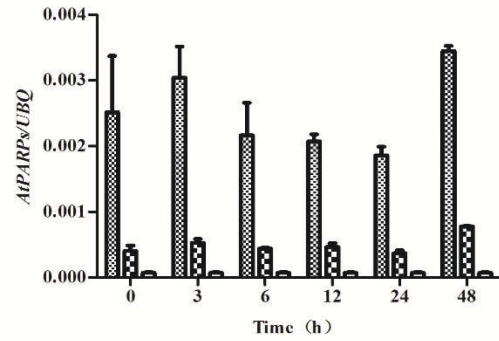

(C)

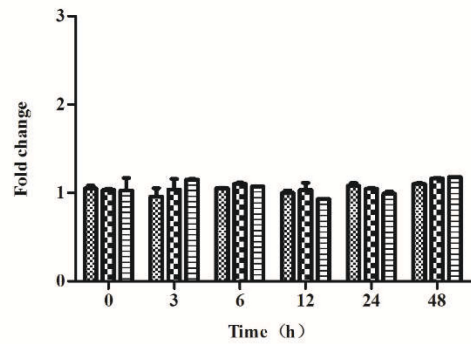

(D)

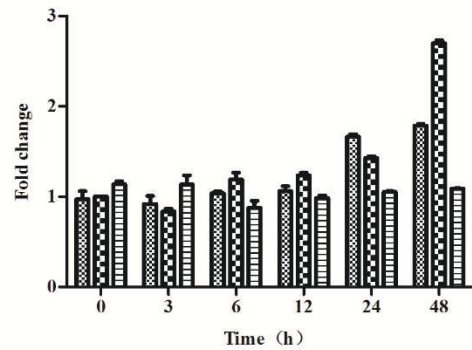

(E)

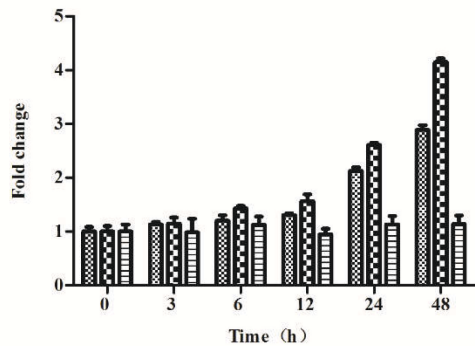

(F)

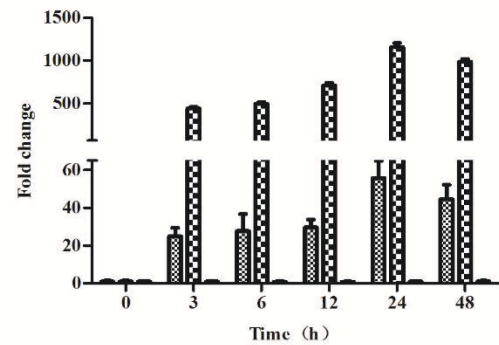

Supplement: Supplementary file 4 — Figure S4 Transcription levels of AtPARPs under normal conditions and genotoxin treatments in seeds and seedlings. (A) Relative expression levels of the AtPARP members in Arabidopsis dry seeds. (B) Expression levels of the AtPARP members in Arabidopsis seedlings after distilled water treatment. (C) Comparison of the expression levels of the AtPARP members in seeds after MMS treatment. (D) Comparison of the expression levels of the AtPARP members in seeds after zeocin treatment. (E) Comparison of the expression levels of the AtPARP members in Arabidopsis seedlings after MMS treatment. (F) Comparison of the expression level of the PARP members in Arabidopsis seedlings after zeocin treatment. Seeds of Col-0 and parp mutants were incubated with distilled water (A), 100 μg/mL MMS (C), or 200 μg/mL zeocin (D) for different time periods. 10-d-old Col-0 and parp mutants seedlings grown on ½ MS plates were sprayed with distilled water (B), 100 μg/mL MMS (E), or 200 μg/mL zeocin (F) for different time periods. Total RNA was extracted from seeds or seedlings and subjected to RT-qPCR analysis. The expression levels of AtPARPs were normalized to that of AtUBQ5. Simultaneous mock experiments were performed by treating plants with distilled water, and fold changes were calculated by normalizing the gene expression levels under treatment to that of the corresponding mock experiment. The results are shown as the means ± SDs from three biological replicates. (PDF 12025 kb) [file 12870_2019_1958_MOESM4_ESM.pdf]

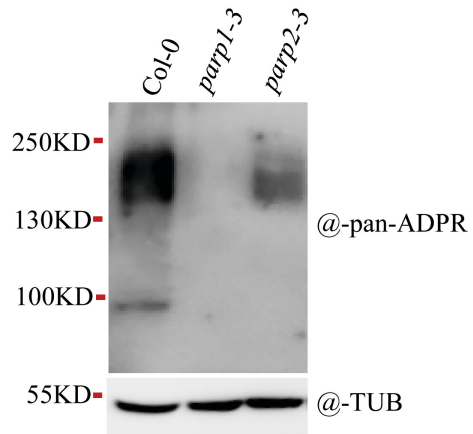

Supplement: Supplementary file 6 — Figure S5 Comparison of PAR signals in Col-0, parp1 and parp2 mutants. 10-d-old seedlings were treated by 200 μg/mL zeocin for 48 h. The total proteins in the seedlings were extracted, blotted and then detected using anti-pan-ADPR reagent. Tubulin was detected using an anti-tubulin antibody to show the protein loading amounts. TUB, tubulin. (PDF 2355 kb) [file 12870_2019_1958_MOESM6_ESM.pdf]

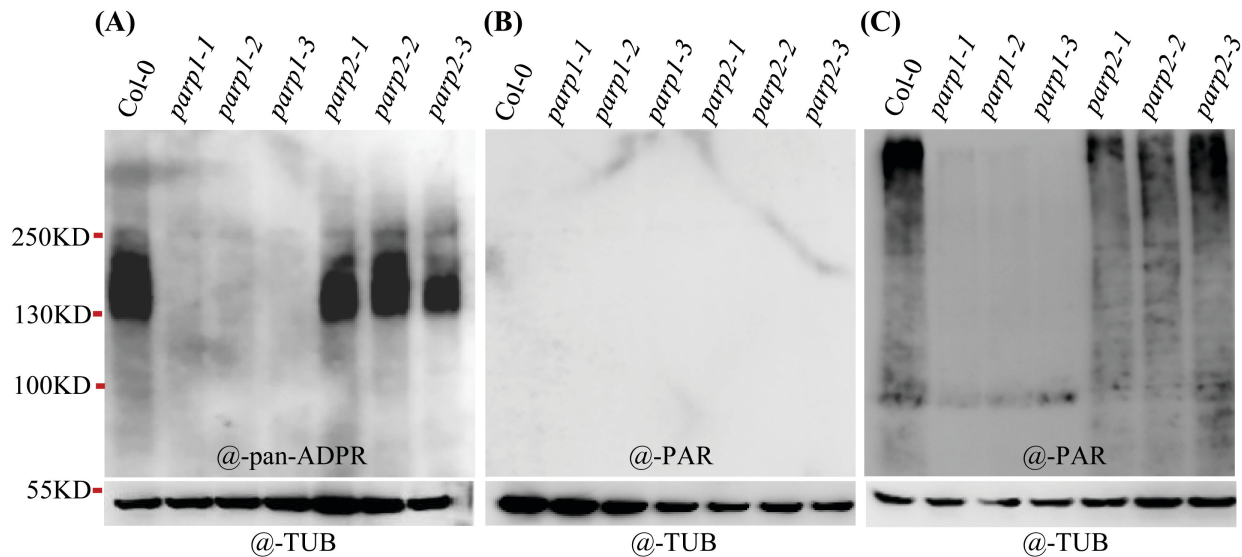

Supplement: Supplementary file 7 — Figure S6 Comparison of the PAR signals detected by anti-pan-ADPR reagent and anti-PAR antibody, respectively. (A) PAR signals in different plants were detected by anti-pan-ADP-ribose binding reagent. (B) PAR signals in different plants were detected by anti-PAR antibody. No signal could be detected on the membrane. (C) PAR signals in different plants were detected by anti-PAR antibody with exogenous NAD+ and activated DNA in the extraction buffer. 0.3 mM NAD+ and 100 nM broken DNA were added into the protein extraction buffer to enhance the PARP catalysis reactions. For (A), (B) and (C), 10-d-old seedlings were treated by 200 μg/mL zeocin for 48 h and the total proteins in the seedlings were extracted and used for western blot. For (B), the samples are the same as those in (A) but detected with anti-PAR antibody. (C), Total proteins were extracted using the same buffer as (A) and (B) except in it 0.3 mM NAD+ and 100 nM broken DNA were added. TUB, tubulin; @-pan-ADPR, anti-pan-ADPR reagent; @-PAR, anti-PAR antibody. (PDF 7691 kb) [file 12870_2019_1958_MOESM7_ESM.pdf]

**(A)**

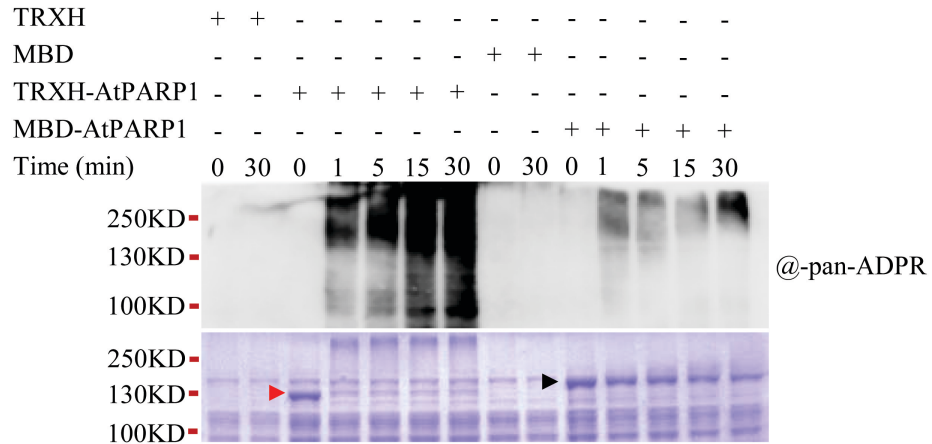

**(B)**

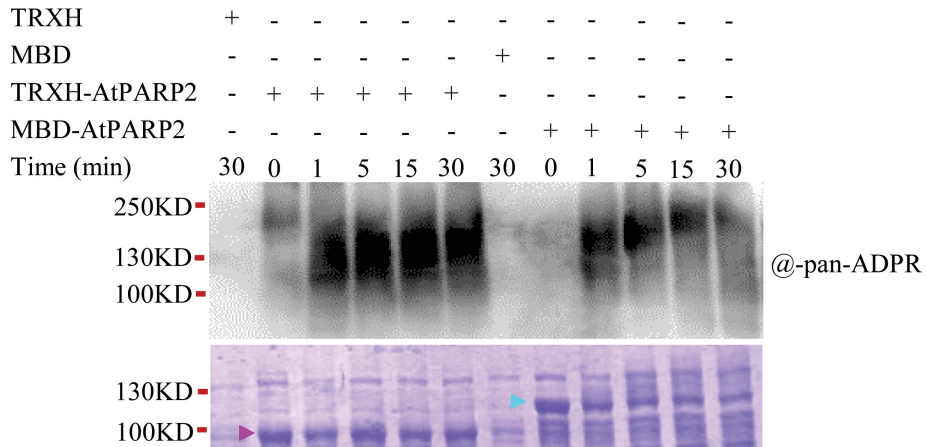

Supplement: Supplementary file 8 — Figure S7 Comparison of the activities of MBD-fused and TRXH-fused recombinant AtPARP proteins. (A) Comparison of the activities of different tag-fused AtPARP1 proteins. (B) Comparison of the activities of different tag-fused AtPARP2 proteins. The purified proteins were incubated with 500 nM DNA and 1 mM NAD+ at 25 °C for different time periods. After the reaction, the proteins were analyzed by immunoblotting with anti-pan-ADPR reagent (the upper panel). Arrows in the bottom Coomassie blue-stained gel indicate the recombinant proteins TRXH-AtPARP1 (red), MBD-AtPARP1 (black), TRXH-AtPARP2 (cyan), and MBD-AtPARP2 (blue). (PDF 7207 kb) [file 12870_2019_1958_MOESM8_ESM.pdf]

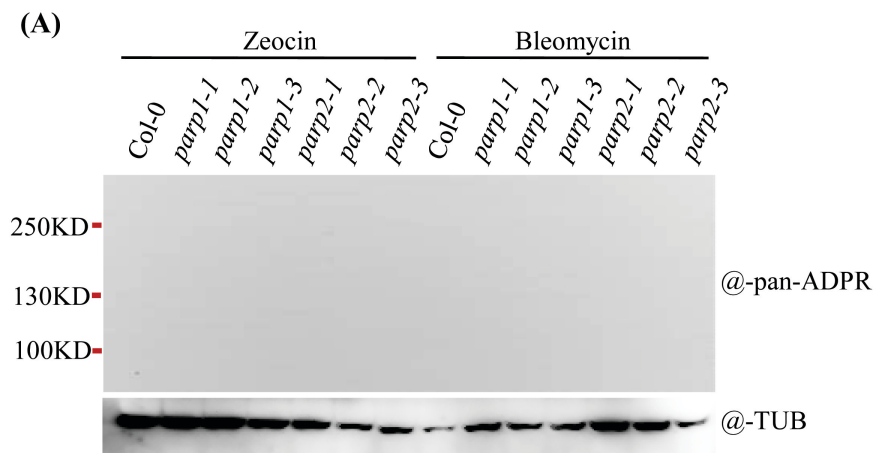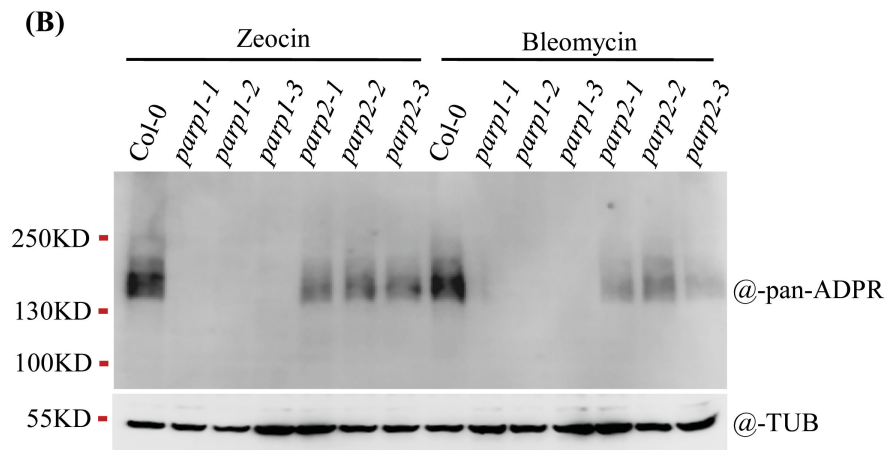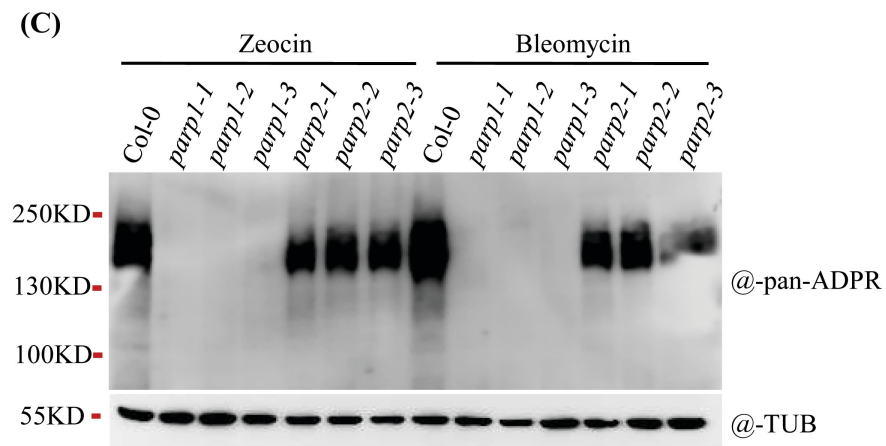

Supplement: Supplementary file 9 — Figure S8 AtPARP1 was responsible for the generation of most of the PAR signals under bleomycin and zeocin treatments. (A) PAR signals in different parp1 and parp2 mutants after mock (H2O) treatment for 24 h and 48 h, respectively. (B) PAR signals in different parp1 and parp2 mutants after 200 μg/mL zeocin or 25 μg/mL bleomycin treatment for 24 h. (C) PAR signals in different parp1 and parp2 mutants after 200 μg/mL zeocin or 25 μg/mL bleomycin treatment for 48 h. 10-d-old seedlings were treated with zeocin or bleomycin for different time periods and then the total proteins were extracted and detected using anti-pan-ADPR reagent. Tubulin was detected using an anti-tubulin antibody to indicate the protein loading amounts. TUB, tubulin; @-pan-ADPR, anti-pan-ADPR reagent. (PDF 9141 kb) [file 12870_2019_1958_MOESM9_ESM.pdf]
