# Supplementary material for: New perspectives on the plant PARP family: Arabidopsis PARP3 is inactive, and PARP1 exhibits predominant poly (ADP-ribose) polymerase activity in response to DNA damage
Source: BMC Plant Biol. 2019 Aug 19;19:364. doi: 10.1186/s12870-019-1958-9 (PMC6701155; doi:10.1186/s12870-019-1958-9)
Supplement: Supplementary file 10 — Table S2 Primers used in this work. (PDF 12294 kb) [file 12870_2019_1958_MOESM10_ESM.pdf]

Table S1. Primers used in this work.

Purpose	Primer name	Sequence (5'-3')
Protein expresion vector construction	PARP1-pET32a-FP (Sal I)	CGAGCTCAAATGGCAAGCCCACATAAGC
	PARP1-pET32a-RP (NotI)	AAGGAAAAAA GCGGCCGCTTCTCTTGTGCTTAAACCTTACTTTC
	PARP1-pMALL-FP (NdeI)	GGGAATTCCATATGATGGCAAGCCCACATAAGC
	PARP1-pMALL-RP (Sal I)	ACGCGTCGACTCATCTCTTGTGCTTAAACCTTACT
	PARP2-pET32a-FP (BamHI)	CGCGGATCCATGGCGAACAAGCTCAAAGTC
	PARP2-pET32a-RP (XhoI)	CCGCTCGAGTTAGTGCTTGTAGTTGAATTTGACTTG
	PARP3-pET32a-FP (SalI)	ACGC GTCGACAAATGAAGGTTCACGAGACAAGATCT
	PARP3-pET32a-RP (NotI)	AAGGAAAAAAGCGGCCGCTCTCTGGTTCGACATCGACTATCTC
	PARP3-pGEX-4T-1-FP (SalI)	ACGCGTCGACAAATGAAGGTTCACGAGACAAGATCT
	PARP3-pGEX-4T-1-RP (NotI)	AAGGAAAAAACTCTGGTTCGACATCGACTATCTC
Point mutation protein expression vector construction	PARP1 (H-C)-RP	TTCGTTAATCGAGAACCACACCATAGGAGCATCTTATTGC
	PARP1 (H-C)-FP	GCAATAAGATGCTCCTATGGTGTGGTTCTCGATTAACGAA
	PARP1 (Y-V)-RP	CTGACAAGGTCAGCAAAGACTATCCCTTTTCCAAACATG
	PARP1 (Y-V)-FP	CATGTTTGGAAAAGGGATAGTCTTTGCTGACCTTGTCAG
	PARP3 (C-H)-RP	TTGAGCTCCGAGACCCATGCCATAAAAGGACCTTATTT
	PARP3 (C-H)-FP	AAATAAGGTCCTTTTATGGCATGGGTCTCGGAGCTCAA
	PARP3 (V-Y)-RP	CTTCTGCAGCTGCATCTGAACAGTATATCGCTCTCCCAAAC
	PARP3 (V-Y)-FP	GTTTGGGAGAGCGATATACTGTTCAGATGCAGCTGCAGAAG
Domain swapping protein expression vector construction	P3-P1 F1-FP	GACGACGACAAGGCCATGATGAAGGTTCACGAGACAAGATCT
	P3-P1 F1-RP	GAAAAACTTATGAGGCTTCTTTTGA
	P3-P1 F2-FP	AGAAGCCTCATAAGTTTTTC CCATTTCAGACCAGTAGCAACC
	P3-P1 F2-RP	CGAGTGCGGCCGCAAGCT TCATCTCTTGTGCTTAAACCTTACTT
	P1-P3 F1-FP	GACGACGACAAGGCCATGATGGCAAGCCCACATAAGC
	P1-P3 F1-RP	CTCTTTTTTGGCTACTTGCTTATTA
	P1-P3 F2-FP	AGCAAGTAGCCAAAAAAGAG CCAATTGATATGGATGATGGAAT
	P1-P3 F2-RP	CGAGTGCGGCCGCAAGCT CTACTCTGGTTCGACATCGACTATC
RT-qPCR	qPARP3-FP	ATGTTTGGGAGAGCGATAGTG
	qPARP3-RP	CCAAGTGATGCTACGGCTAATA
	qPARP1-FP	GATAAACCTCCGAGAGGGAAACA
	qPARP1-RP	CACAGGGAACAGTCACATCA
	qPARP2-FP	CTCACTAACTGGGCTGGTATTT
	qPARP2-RP	TCGCACTCTTGGAGAACATATC
	qUBQ5-FP	AATGTGAAGGCGAAGATCCAAGAC
	qUBQ5-RP	AGACGGAGGACGAGATGAAGC
Protein activity assay	Palindromic DNA oligo	GGAATTCCGGAATTCC
