# Supplementary material for: New perspectives on the plant PARP family: Arabidopsis PARP3 is inactive, and PARP1 exhibits predominant poly (ADP-ribose) polymerase activity in response to DNA damage
Source: BMC Plant Biol. 2019 Aug 19;19:364. doi: 10.1186/s12870-019-1958-9 (PMC6701155; doi:10.1186/s12870-019-1958-9)
Supplement: Supplementary file 11 — Table S3 List of genes used for phylogenetic analysis of the PARP1/2/3 subfamily in the PARP family. (PDF 10506 kb) [file 12870_2019_1958_MOESM11_ESM.pdf]

Table S2. List of genes used for phylogenetic analysis of PARP1/2/3 subfamily in PARP family.

Species name	Gene name	Protein id	Gene pacid	Database
Arabidopsis thaliana	PARP3	AT5G22470.1	19672134	Phytozome
Capsella rubella	PARP3	Carubv10002547m	20910465	Phytozome
Brassica rapaFPsc	PARP3	Brara.J01467.1.p	30613201	Phytozome
Carica papaya	PARP3	evm.model.supercontig_32.96	16417480	Phytozome
Theobroma cacao	PARP3	Thecc1EG041443t1	27427780	Phytozome
Glycine max	PARP3	Glyma.12G088300.1.p	30548414	Phytozome
Glycine max	PARP3	Glyma.11G184100.1.p	30529013	Phytozome
Phaseolus vulgaris	PARP3	Phvul.011G093100.1.p	37156572	Phytozome
Medicago truncatula	PARP3	Medtr4g053530.1	31107311	Phytozome
Cucumis sativus	PARP3	Cucsa.385080.1	16982517	Phytozome
Vitis vinifera	PARP3	GSVIVT01036149001	17841171	Phytozome
Nelumbo nucifera		NNU_16940-RA, 016940		http://lotus-db.wbgcas.cn
Mimulus guttatus	PARP3	Migut.D02355.1.p	28927533	Phytozome
Solanum tuberosum	PARP3	PGSC0003DMP400013102	37467110	Phytozome
Populus trichocarpa	PARP3	Potri.004G184100.1.p	37232163	Phytozome
Oryza sativa	PARP3	LOC_Os02g32860.1	33139988	Phytozome
Sorghum bicolor	PARP3	Sobic.004G164800.1.p	32746215	Phytozome
Picea abies		125674g0010		ConGenIE
Ginkgo biloba		02125		GigaDB
Selaginella moellendorffii	PARP3	73333	15406099	Phytozome
Physcomitrella patens	PARP3	Pp3c1_22640V3.1.p	32971758	Phytozome
Homo sapiens	PARP1	ENST00000366794.9		Ensembl
Mus musculus	PARP1	ENSMUST00000027777.11		Ensembl
Drosophila melanogaster	PARP	FBtr0113885		Ensembl
Caenorhabditis elegans	PARP1	Y71F9AL.18a		Ensembl
Mycena alexandri	PARP	1092715		MycoCosm
Mycena alexandri	PARP	1127241		MycoCosm
Sarcoscypha coccinea	PARP	423918		MycoCosm
Sarcoscypha coccinea	PARP	510097		MycoCosm
Homo sapiens	PARP2	ENST00000250416.9		Ensembl
Mus musculus	PARP2	ENSMUST00000036126.5		Ensembl
Homo sapiens	PARP3	ENST00000398755.7		Ensembl
Mus musculus	PARP3	ENSMUST00000112479.8		Ensembl
Caenorhabditis elegans	PARP2	E02H1.4		Ensembl
Caenorhabditis elegans	TANK-1/PARP5	ZK1005.1a		Ensembl
